# CRISPR‐DNA Polymerase Assisted Targeted Mutagenesis for Regulable Laboratory Evolution

**DOI:** 10.1002/advs.202511448

**Published:** 2025-09-23

**Authors:** Shuaili Chen, Xiangdi Chen, Yifan Peng, Qinghua Li, Jingwen Zhou, Jianghua Li, Guocheng Du, Jian Chen, Guoqiang Zhang

**Affiliations:** ^1^ Science Center for Future Foods Jiangnan University 1800 Lihu Road Wuxi Jiangsu 214122 China; ^2^ School of Biotechnology and Key Laboratory of Industrial Biotechnology of Ministry of Education Jiangnan University Wuxi 214122 China; ^3^ Jiangsu Province Basic Research Center for Synthetic Biology Jiangnan University Wuxi 214122 China

**Keywords:** CRISPR, laboratory evolution, regulable mutagenesis, T5/T7 DNA polymerase, targeted hypermutation

## Abstract

Targeted hypermutation tools are useful for engineering proteins and pathways, and exploring the evolutionary landscapes. However, existing targeted hypermutation tools for genomic loci mostly exhibit restricted mutation windows and limited mutational types. Here, by integrating mutagenic, high‐processivity bacteriophage T5 or T7 DNA polymerases (DNAPs) with CRISPR‐Cas9, the study develops an in vivo mutagenesis system that enables all possible types of nucleotide substitutions and an expanded mutation window of up to 2 kilobases, achieving a maximum mutation rate 1.1 × 10^6^‐fold higher than wild‐type *Escherichia coli*. Through MS2‐mediated recruitment of T5 or T7 DNAP for co‐localization with nickase nCas9, off‐target rate is reduced by up to 96.8% without compromising on‐target rate. Further benefiting from the dTnpB‐based transcriptional repression system, the mutagenesis process can be properly regulated during continuous evolution. Finally, the CRISPR‐TDNAP‐assisted targeted mutagenesis for regulable laboratory evolution (CTRLE) confers cellular triple‐antibiotic resistance in 8 days, and enhances the efficiency of the twin‐arginine translocation pathway by over threefold in 6 days. Furthermore, CTRLE proves effective in *Bacillus subtilis* and *Kluyveromyces lactis*, yielding targeted mutation rates 1.2 × 10⁵‐fold and 5 × 10⁷‐fold higher than host backgrounds, respectively. Collectively, CTRLE provides an efficient and universal way to accelerate the continuous evolution of different microbial cells.

## Graphical abstract

This study develops an in vivo targeted mutagenesis system based on CRISPR‐guided bacteriophage T5 or T7 DNA polymerases. This system can enable all possible types of substitution mutations within an expanded mutagenesis window in *Escherichia coli* and exhibit application potential in different microorganisms, thus accelerating the evolution of microbial cell factories.

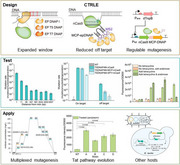

## 1 Introduction

Laboratory evolution is a powerful approach for obtaining improved biomolecules and biological systems for biotechnology, pharmaceutical, and industrial applications. It is a traditional strategy of accumulating beneficial mutations through genetic diversity in vivo and the expected selection pressure. However, due to the low mutation rate (10^−9^–10^−11^ per base per generation) of living organisms, it usually requires crossing a long time scale to obtain the desired traits, which limits the evolution efficiency.^[^ ^1^ ^]^ Traditional directed evolution depends on the mutation libraries generated in vitro, and involves circular operation of DNA transformation and selection, which is labor‐intensive and limits the evolution scale. In vivo mutagenesis technology circumvents these issues by utilizing mutagenic factors to elevate the mutation rate and avoid low transformation efficiency, enables continuous processes because of automatic diversification and selection.

In vivo mutagenesis tools have been developed for targeted diversification of both plasmid‐borne and chromosomal genes. Targeted hypermutation tools mainly relying on error‐prone replication to produce mutation directly within gene of interest on plasmid, such as phage‐assisted continuous evolution (PACE) technology^[^ ^2^ ^]^ on the basis of virus replication, the orthogonal DNA replication system in yeast (OrthoRep)^[^ ^3^ ^]^ and prokaryotic microorganisms^[^ ^4^, ^5^ ^]^ and targeted artificial or engineered DNA replisome (TADR and T7‐ORACLE)^[^ ^6^, ^7^ ^]^ that replicated plasmid with low fidelity. MutaT7 consisting of base deaminases and T7 RNA polymerase is also a frequently‐used mutagenesis tool to mutate the genes upstream of the T7 promoter sequence in a plasmid.^[^ ^8^ ^]^ Targeted hypermutation across plasmid contributes to the continuous evolution of proteins with the desired functions by cloning their coding sequences into specialized vectors such as phage DNA, the specific plasmids, or locations downstream of a T7 promoter. However, this limits the in situ evolution of some proteins that require to be expressed endogenously and assessed functionally to maintain the compatibility with host context.

Targeted hypermutation tools for genomic loci addresses these issues via single‐stranded DNA (ssDNA)‐ or RNA‐mediated recombination and clustered regularly interspaced short palindromic repeat (CRISPR)‐Cas tools. Hypermutation tools based on recombination involve ssDNA or RNA generation with errors through a retron library,^[^ ^9^ ^]^ error‐prone reverse transcriptase,^[^ ^10^ ^]^ or T7 RNA polymerase,^[^ ^11^, ^12^ ^]^ and introduce mutations mostly within a 1‐kb window. One prevalent strategy of CRISPR‐mediated tools is to fuse base deaminases with Cas9 or Cas12 variants, the resultant base editors enable a narrowed editing widow (<43 nt) with the limited nucleotide substitutions at specific genome locus relying on the guidance of guide RNAs.^[^ ^13^, ^14^, ^15^ ^]^ Another similar fusion, EvolvR where error‐prone DNA polymerase I (PolI3M) is fused with the Cas9 nickase (nCas9), can generate 12 nucleotide substitutions in both bacteria and yeast via PolI repairing the Cas9 nick site, which has be extensively used for plasmid‐ and genome‐wide targeted hypermutation to engineer enzymes with desired properties.^[^ ^16^, ^17^ ^]^ Despite leading to the increased mutation rate up to 7 770 000‐fold using varying mutagenic PolIs, EvolvR introduced mutations in a restricted window (<350 nt) and usually requires to design multiple single guide RNAs (sgRNAs) to cover the entire gene along with the elevated off‐target mutation rate, which hinders the rapid and efficient diversification of targeted genes. Furthermore, a hypermutation tool usually functions well in only one specific host due to its dependence on host context, which limits in situ mutagenesis in different hosts. In addition, precise regulation of mutator expression can conditionally turn on the mutagenesis system, which enables a strict and controllable mutagenesis process. Therefore, developing a hypermutation tool with a widened editing widow, low off‐target mutation rate, and rigorously regulated expression is necessary to accelerate the evolution of genomic proteins and microbial chassis.

In light of the modularity of CRISPR‐based tools, we sought to expand the mutagenesis region adjacent to the Cas protein binding site via incorporating error‐prone bacteriophage DNA polymerase (T5 or T7 DNAP) that synthesizes DNA with high processivity. Two more active versions, CRISPR‐based T5 and T7 DNAPs, enable a significantly increased mutagenesis window and were further optimized for decreased off‐target rate via MS2‐mediated recruitment, and precisely spatiotemporal‐controllable mutagenesis through a transcriptional regulation system based on deactivated TnpB (dTnpB). This system was termed as CRISPR‐TDNAP‐assisted targeted mutagenesis for regulable laboratory evolution (CTRLE). Furthermore, 3 target loci distributed across the genome were continuously evolved simultaneously in 8 days using CTRLE, which conferred an *Escherichia coli* (*E. coli*) strain increased rifampicin, spectinomycin, and streptomycin resistance from 0 to 200 µg mL^−1^. Additionally, the secretion efficiency of periplasmic proteins was increased by threefold in *E. coli* through continuous evolution for 6 days. Moreover, CTRLE also exhibited significantly increased targeted mutagenesis capacity in *Bacillus subtilis* (*B. subtilis*) and *Kluyveromyces lactis* (*K. lactis*). As a conclusion, CTRLE with an expanded mutation window, multiplexed mutagenesis capacity, is conducive to increase the efficiency of continuous evolution.

## 2 Results

### 2.1 Construction of nCas9‐T5/7 DNAP Mutator in *E. coli*

**Figure 1:**
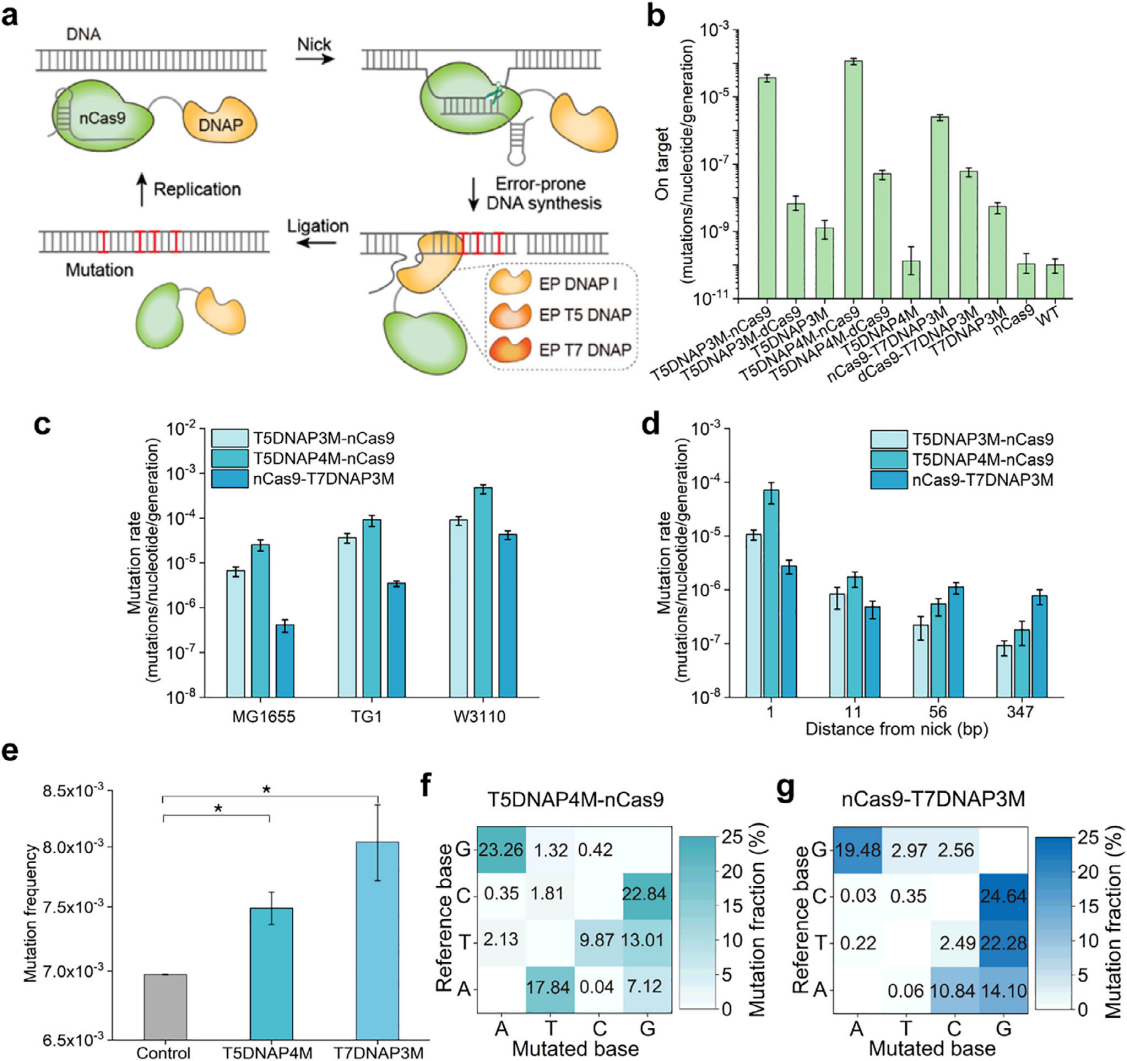
Design of nCas9‐guided T5/7 DNAP targeted mutator in *E. coli*. a) Working principle of nCas9‐guided DNAP targeted mutator. b) The mutation rate generated by three mutagenic polymerases (T5DNAP3M, T5DNAP4M, and T7DNAP3M) expressed alone and fused with nCas9 or dCas9 at 1 bp from the nick in *E. coli* TG1. Data are the mean ± 95% confidence intervals from six biologically independent replicates (*n *= 6). c) The mutation rate of CRISPR‐guided T5DNAP3M, T5DNAP4M, and T7DNAP3M in different strains of *E. coli* (*n *= 4, Data are presented as mean ± 95% confidence intervals). d) The mutation rate resulted from T5DNAP3M‐nCas9, T5DNAP4M‐nCas9, and nCas9‐T7DNAP3M at 1, 11, 56, and 347 bp from nick (*n *= 4, Data are presented as mean ± 95% confidence intervals). e) Average mutation frequency of T5DNAP4M‐nCas9 and nCas9‐T7DNAP3M targeted to whole plasmid (*n *= 3, Data are presented as mean ± SD). *P < 0.05, two‐sided student's *t*‐test. Mutagenic spectra of T5DNAP4M‐nCas9 (f) and nCas9‐T7DNAP3M (g).

Based on the fusion of CRISPR‐guided nCas9 and error‐prone DNAP, diversification of all nucleotides can be generated at a targeted loci, such as the EvolvR (**Figure** **1**a).^[^ ^16^ ^]^ It was hypothesized that fusing a more processive DNAP to the nickase would expand the mutagenesis region, which can decrease the number of sgRNAs designed especially for the continuous evolution of long genes. PolI from the host itself may be involved in host genome replication leading to increased off‐target rate, an error‐prone DNAP from other organisms may avoid this limitation. PolI with a processivity of 15 to 20 nucleotides is the prototype member of family A of DNAPs which participates in DNA replication and repair in eukaryotes, bacteria, and bacteriophages.^[^ ^18^, ^19^ ^]^ T7 DNAP with its processivity factor *E. coli* thioredoxin is a typical member of phage family A of DNAPs and essential for linear genome replication of phage T7, which has been well‐characterized.^[^ ^20^, ^21^ ^]^ Considering that nick‐translating activity of PolI plays a crucial role in EvolvR,^[^ ^22^ ^]^ T5 DNAP not only perform DNA synthesis in a highly processive mode, but also possesses strand‐displacing activity for nicked DNA templates like DNA PolI, which makes it another candidate.^[^ ^23^, ^24^ ^]^

On the basis of previous reports, three distinct mutagenic DNA polymerase variants were utilized as mutators: a T5 DNAP variant carrying three amino acid substitutions D164A, E166A, and A593R (T5 DNAP3M); an additional T5 DNAP variant incorporating four mutations D164A, E166A, A593R, and I308V (T5 DNAP4M); and an exonuclease‐deficient T7 DNAP variant harbouring three substitutions Y64C, F120L, and S399T (T7 DNAP3M).^[^ ^6^, ^25^ ^]^ A linker containing 23 amino acids was employed to connect nCas9 to the carboxyl terminus of T5DNAP3/4M, since fusing proteins to the carboxyl terminus of T5 DNAP did not affect its activity.^[^ ^6^ ^]^ Due to the close proximity of the T7DNAP3M active site to the carboxyl terminus,^[^ ^6^ ^]^ nCas9 was fused with the amino terminus of T7DNAP3M to prevent interference with T7 DNAP polymerization activity, similarly. To test this possibility, the fusion mutators were expressed under the control of the tetracycline‐inducible promoter P_tet_ using the multiple‐copy‐number plasmid pET28a(+) (Figure S1, Supporting Information). Spectinomycin resistance gene expression cassette containing a premature termination codon (L33Stop) was constructed in low‐copy‐number plasmid pACYCDuet‐1 to serve as a reporter (Figure S1, Supporting Information). These constructs were performed the fluctuation test for mutation rate determination using the sgRNA target generating a nick at 1 nucleotides from 5′ of the nonsense mutation (Figure 1a). As shown in Figure 1b, expressing T5DNAP3M, T5DNAP4M and T7DNAP3M alone showed a 12 ‐ fold, 0.31‐fold, and 53‐fold increase in mutation rate at 1 bp from nick in comparison to the wild‐type stain (1.01 × 10^−10^), and their fusion with dCas9 further improved the mutation rate, whereas their fusion with nCas9 showed substantial levels of base substitution activity. The expression of T5DNAP3M‐nCas9, T5DNAP4M‐nCas9, and nCas9‐T7DNAP3M increased the mutation rate at the target site by 1.07 × 10^5^‐fold, 7.08 × 10^5^‐fold, and 2.76 × 10^4^‐fold, respectively (Figure 1b).

To prove the practicability of the mutators, T5DNAP3/4M‐nCas9 and nCas9‐T7DNAP3M were tested in two other hosts of *E. coli* strains (W3110 and MG1655). The results showed that the same trend in mutation level was observed in different strains, with T5DNAP4M resulting in the highest mutation rate, followed by T5DNAP3M, and the last T7DNAP3M (Figure 1c). Among the above *E. coli* strains, TG1 was selected as the host for conducting in vivo mutagenesis and continuous evolution experiments, due to its moderate mutagenesis efficiency and the fastest growth rate (Figure 1c). In summary, all three mutators can effectively increase the mutation rate on target sites in three strains of *E. coli*.

### 2.2 nCas9‐Guided T5/7 DNAP Mutator with Hypermutation Rate and Widened Window

In order to evaluate the editing window, a series of sgRNAs is designed to target different positions from the *aad*A premature termination codon. While the mutational trends of T5DNAP3M‐nCas9 and T5DNAP4M‐nCas9 were broadly similar across the 350 bp region, T5DNAP4M‐nCas9 resulted in a higher overall average mutation rate (Figure 1d), which was also consistent with the result mentioned above. Therefore, the T5DNAP4M‐nCas9 system was used for further research. Although the mutation rate of T5DNAP3M‐ and T5DNAP4M‐nCas9 decreased sharply within 11 bp of the nick, they exhibited a gradual decline across the 11–347 bp range. Similarly, the mutation rate of nCas9‐T7DNAP3M was also affected by the length from the nick. The mutation rates of the three mutators at 347 bp exceeded 9.1 × 10^−8^ per base per generation (Figure 1d). To further increase the mutation rate, an enhanced nCas9 (enCas9, nCas9 K848A/K1003A/R1060A) with lower non‐specific DNA affinity was applied and tested. T5DNAP4M‐enCas9 and enCas9‐T7DNAP3M exhibited over one‐fold higher mutation rates compared to T5DNAP4M‐nCas9 and nCas9‐T7DNAP3M (Figure S2a, Supporting Information). T5DNAP4M‐enCas9 and enCas9‐T7DNAP3M mutators were observed mutations at a rate of approximately 1 × 10^−7^ per base per generation at 2000 bp from nick site, demonstrating that the mutagenesis could cover 2000 bp (Figure S2b,c, Supporting Information). Therefore, the highly processive nature of T5/7 DNAP may permit access to a wider mutagenesis window was achieved by nCas9‐T5/7 DNAP mutator due to the highly processive nature of T5/7 DNAP.

Cultures through mutagenesis of 200 generations were used for next‐generation sequencing to analyze the mutational spectrum. Compared to the pre‐evolutionary sample, the average mutation frequency of T5DNAP4M and T7DNAP3M was elevated in the 2‐kb targeted sequence, indicating that mutations occurred in the targeted region of the plasmid (Figure 1e). Both T5DNAP4M and T7DNAP3M could achieve 12 types of substitution mutations with a different bias for transitions (G to A for T5DNAP4M and A to G and G to A for T7DNAP3M) and transversions (A to T, T to G, and C to G for T5DNAP4M and T to G and C to G for T5DNAP4M) (Figure 1f,g). In summary, nCas9‐guided T5/7 DNAP targeted mutator has the potential to mutate long genes and access all types of nucleotide substitutions.

### 2.3 Recruitment Protein MCP‐Associated Mutator with Lower Off‐Target Mutagenesis

**Figure 2:**
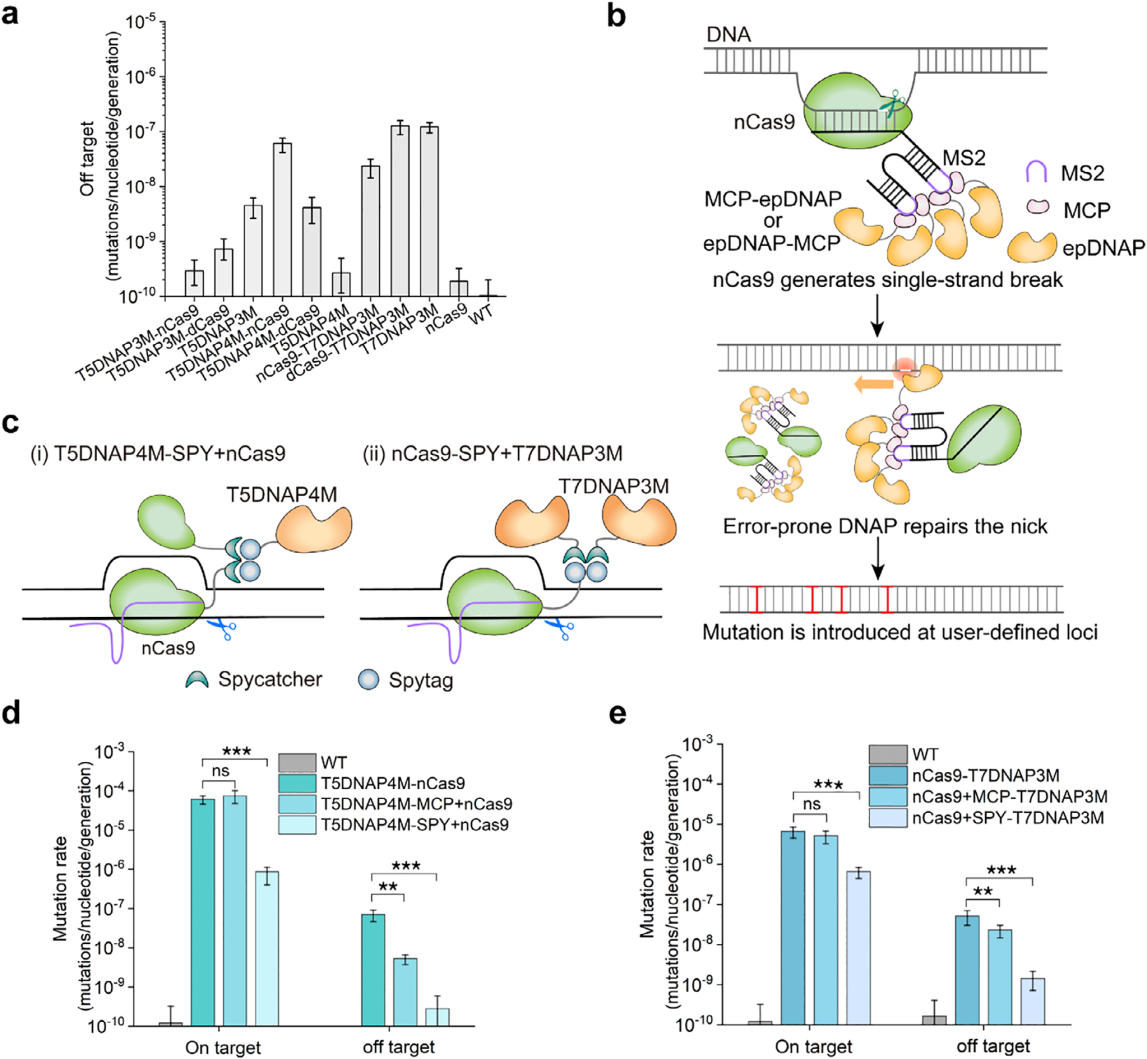
Recruitment protein MCP‐associated mutator with lower off‐target mutagenesis. a) Off‐target mutation rates of T5DNAP3M alone, T5DNAP4M alone, and T7DNAP3M alone, their respective fusion with nCas9 or dCas9, nCas9 alone, and pET28a (WT) containing a sgRNA targeting the genome *dbpA* (*n* = 6, Data are presented as mean ± 95% confidence intervals). b) A diagram of MS2 aptamer‐mediated recruitment of error‐prone DNAP (epDNAP) to nCas9 resulting in mutations at the target locus. MCP‐fused error‐prone polymerase (epDNAP) was recruited to the sgRNA incorporated two MS2 hairpin loops relying on the interaction between the MCP dimer and the MS2 aptamer. For the T5DNAP4M‐MCP+nCas9 construct, MCP was fused via a 23‐amino acid linker to the C‐terminus of T5DNAP4M, while nCas9 was expressed separately downstream. In the case of nCas9+MCP‐T7DNAP3M, MCP was fused to the N‐terminus of T7DNAP3M, with nCas9 being expressed independently upstream. Pink and brown patterns represent MCP and epDNAP, respectively. c) Schematic diagram of recruiting T5DNAP4M (i) and T7DNAP3M (ii) to nCas9 using the SpyTag/SpyCatcher system. For the T5DNAP4M‐SPY+nCas9 construct, T5DNAP4M C‐terminally connected to dual SpyTag repeats through a 23‐amino acid linker, with nCas9 fused to SpyCatcher's C‐terminus. For the nCas9‐SPY+T7DNAP3M construct, nCas9 was fused to the N‐terminus of dual SpyTag repeats, while T7DNAP3M connected to SpyCatcher's C‐terminus via a 23‐aa linker. The on‐target (1 bp) and off‐target mutation rate of T5DNAP4M (d) and T7DNAP3M (e) using the strategy of MS2‐ or SPY‐mediated recruitment or not (*n*= 4, Data are presented as mean ± 95% confidence intervals). One nucleotides from 3′ of the nick site was defined as position (+)1 bp. *P < 0.05, **P < 0.01, ***P < 0.001; two‐sided student's *t‐*test.

Off‐target mutagenesis may affect host fitness, leading to competitive selection evasion or false positives during screening. Lowering off‐target mutagenesis is necessary for directed evolution, especially in continuous evolution experiments. To begin with, the off‐target mutation rate was determined using a sgRNA targeting the genome *dbp*A encoding a fitness‐neutral RNA helicase. As shown in **Figure** **2**a, T5DNAP3M or T7DNAP3M fused to nCas9 showed a reduced off‐target mutation rate than individual expression, whereas T5DNAP4M fused to nCas9 showed substantially increased off‐target levels, which may be resulted from some impact on the functional DNAPs when directly fused to functional nCas9.

It was hypothesized that protein recruitment may increase the flexibility of mutator elements to influence off‐target mutagenesis. In order to recruit DNA polymerase to the target locus, we used bacteriophage MS2 coat protein (MCP) fused with T5DNAP4M or T7DNAP3M that generated a higher mutation rate, and introduced two MS2 hairpin loops recruiting two MCP dimers into the stem loops of a single sgRNA (Figure 2b), inspired by the previously reported design of the CRISPR‐X hypermutation system.^[^ ^26^, ^27^ ^]^ As shown in Figure 2d,e, MS2‐associated recruitment of either T5DNAP4M or T7DNAP3M contributed to a comparable mutagenesis level to direct fusion with nCas9 at the target locus, and exhibited a decrease of 96.8% and 54.8% in off‐target mutagenesis, respectively. T5DNAP4M and T7DNAP3M exhibited off‐target mutation rates approximately 43‐ and 215‐fold higher than that of the wild‐type strain (approximately 1.08 × 10^−10^ mutations/bp/generation). In addition, the SpyTag/SpyCatcher split anchoring system was also taken as another alternative solution.^[^ ^28^, ^29^ ^]^ It was observed that both T5DNAP4M and T7DNAP3M recruited by the SPY system exhibited a considerable decrease off‐target mutation rate, however, with significantly lower targeted mutagenesis as well (Figure 2c–e). These results demonstrated that the co‐localization strategy of mutator elements could be used to adjust the on‐ and off‐target mutation rate, which may be due to the changes in space proximity and overall flexibility of mutator.

### 2.4 Spatiotemporal Switch Using dTnpB for In Vivo Regulable Mutagenesis

Uncontrollable mutator systems may lead to undesirable mutagenesis that occurs outside or inside of targeted sequences identified as positives, which makes it difficult to isolate the desired mutants after completing continuous evolution experiments. In this study, although the promoter P_tet_ was observed no leaky expression when expressing enhanced green fluorescent protein (EGFP) alone, it still resulted in a significantly elevated mutation rate when expressing mutators (T5DNAP4M and T7DNAP3M) in the absence of tetracycline (Figure S3, Supporting Information). Therefore, the more rigorous control of mutator expression was needed to suppress leakage expression. TnpB‐based genome editing system,^[^ ^30^ ^]^ due to its orthogonal functionality to Cas9 nuclease, was employed to control the transcription of the mutator cassette. The catalytically dead *TnpB* (TnpB D191A) of *Deinococcus radiodurans ISDra2* was placed downstream of the arabinose‐induced promoter P_ara_, and assembled with its guide RNA (gRNA) targeting promoter P_tet_ into the plasmid. The fluorescence intensity exhibited a concentration‐dependent decrease with increasing arabinose concentration, which demonstrated the effectiveness of dTnpB for repressing EGFP transcription (Figure S4, Supporting Information).

**Figure 3:**
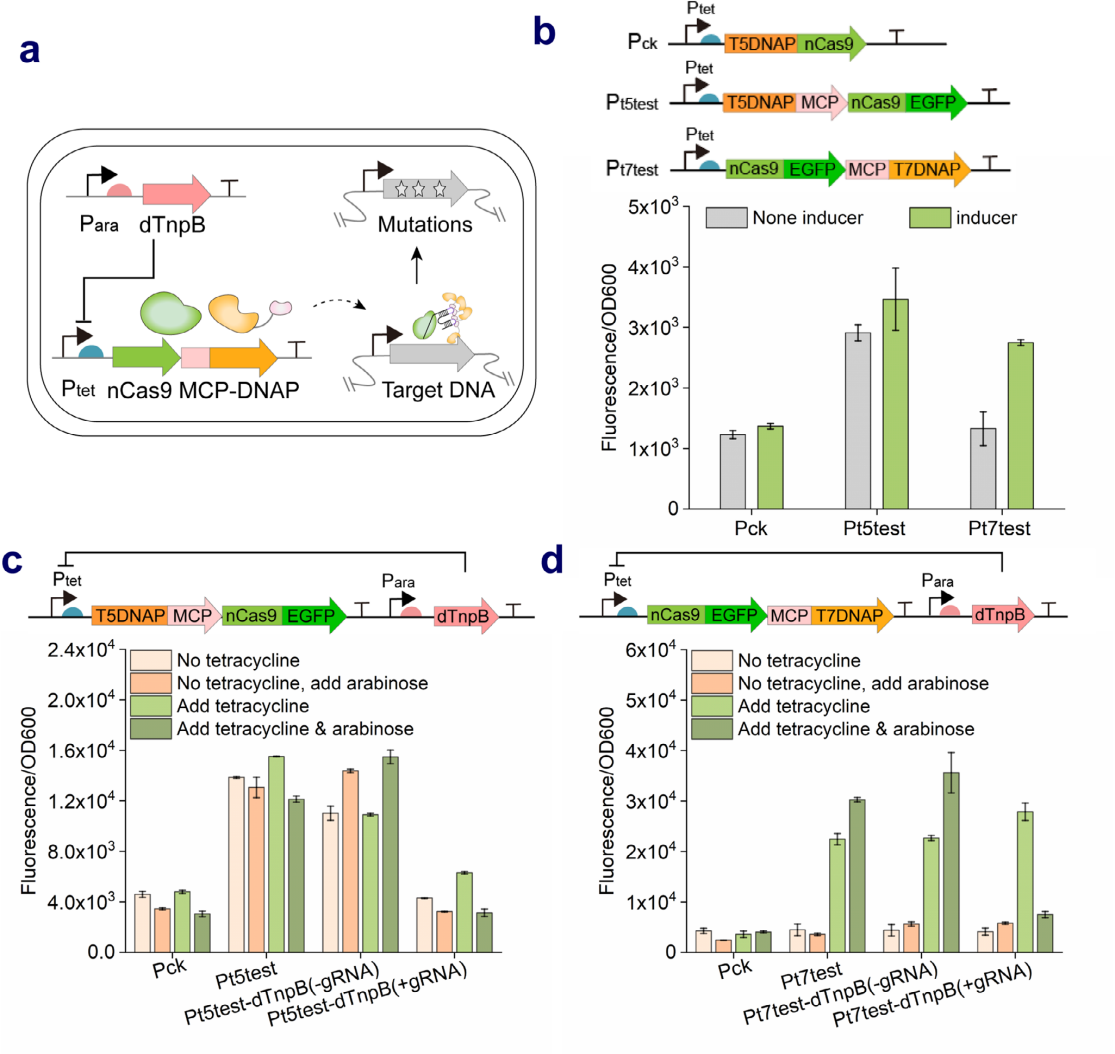
Regulable nCas9‐guided DNAP targeted mutator under dTnpB‐based transcriptional repression. a) Schematic representation of the dTnpB‐mediated transcriptional regulation system for controlling mutator activity, enabling precise ON/OFF switching of mutagenesis. In the absence of arabinose, the P_tet_ promoter allows for the leaking or inducible expression of mutator (including nCas9 and MCP‐epDNAP fusion) leading to producing mutations at specific sequences. When arabinose is available, the dead mutant dTnpB that combines with its gRNA targeting P_tet_ promoter is expressed under the control of P_ara_ promoter, and then binds to the P_tet_ promoter region, thus inhibiting the transcription of the downstream mutator‐encoding sequence to reduce undesired mutagenesis. b) Expression profiles of the *E. coli* TG1 strain co‐transformed Pt5test or Pt7test plasmid with Ptarget plasmid after fermentation for 10 hours in the shake flask. Assessment of dTnpB bearing gRNA or no gRNA and their capacity to repress mutator cassette expression as determined by EGFP fluorescence of Pt5test‐dTnpB (c) and Pt7test‐dTnpB (d) strains upon addition of tetracycline and/or arabinose (*n* = 3, data are shown as mean ± SD).

Next, EGFP was fused into the mutator cassette for constructing plasmid Pt5test and Pt7test to characterize the expression profiles of mutators (**Figure** **3**a,b). Pt5test and Pt7test showed obvious and modest EGFP leakage without a tetracycline inducer, respectively. In order to investigate the regulatory effects on mutator expression, enlarged suppression was determined under tetracycline‐induced conditions. As shown in Figure 3c,d, the fluorescence intensity of Pt5test‐ and Pt7test‐dTnpB with dTnpB gRNA significantly decreased to a level comparable to that of the wild type in the presence of arabinose, which suggested the dTnpB‐based transcriptional repression system effectively suppressed mutator leakage. Notably, in the absence of arabinose, the EGFP expression level of Pt7test‐dTnpB with dTnpB gRNA was comparable to that of Pt7test with tetracycline. However, expression repression was still observed for Pt5test even in the absence of arabinose, despite the presence of 50 ng mL^−1^ tetracycline, suggesting that higher doses of tetracycline inducers may compensate for the mutation rate deficit and offered a range of targeted mutation rates during mutagenesis. Overall, these results indicated that the mutagenic state induced by the nCas9‐guided DNAP targeted mutator can be effectively attenuated through dTnpB‐mediated transcriptional repression of mutator gene expression, establishing a precisely controllable spatiotemporal mutagenesis system. Finally, in this study, the CTRLE system has the advantages of a high targeted mutation rate, a low off‐target rate, a wide window, and controllability.

### 2.5 CTRLE ‐Assisted Multiple Loci Evolution for Cellular Phenotypic Diversification

**Figure 4:**
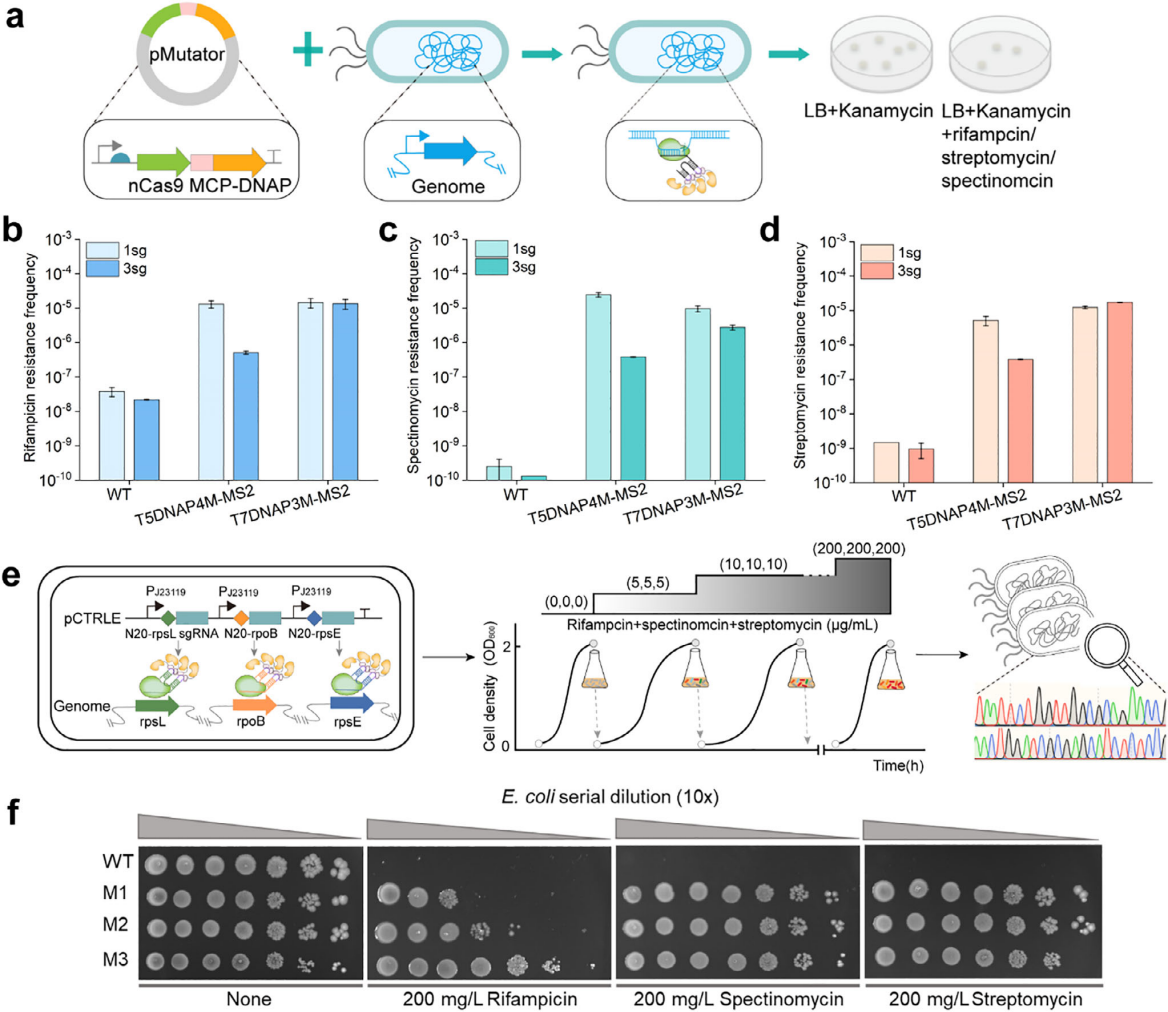
Application of CTRLE for multiplexed in situ mutagenesis. a) Workflow of CTRLE producing mutations at the targeted genomic locus and evaluation of drug resistance frequency in *E. coli* TG1. Rifampicin (b), spectinomycin (c), and streptomycin (d) resistance frequency produced by CTRLE (T5DNAP4M‐ or T7DNAP3M‐MS2) that carried a single sgRNA cassette (1sg) targeting *rpoB*, *rpsE*, or *rpsL* and three tandem sgRNA cassettes (3sg) targeting *rpoB*, *rpsE*, and *rpsL* simultaneously in *E. coli* (*n* = 3, Data are presented as mean ± SD). e) Schematic of strategy to simultaneously evolving rpoB, rpsE, and rpsL for obtaining rifampicin‐spectinomycin‐streptomycin resistant strains. The *rpoB*, *rpsE*, and *rpsL* genes were continuously mutagenized by T5DNAP4M‐ or T7DNAP3M‐MS2. Growth‐based selection for improved rifampicin‐spectinomycin‐streptomycin resistance was achieved via passaging cultures upon the addition of increasing concentrations of rifampicin, spectinomycin, and streptomycin antibiotics. f) Mutant and wild‐type (WT) strains were serially diluted (10×) with PBS buffer, and aliquots (1 µL) of cell suspensions from each dilution series were grown on antibiotic‐containing agar plates at 37 °C for 9 h.

To evaluate the utility of CTRLE for evolving genomic locus, it was applied to the in situ mutagenesis of three genes associated with antibiotic resistance, including *rpoB* gene (encoding the RNA polymerase β subunit),^[^ ^31^ ^]^ *rpsL* gene (encoding ribosomal protein S12),^[^ ^32^ ^]^ and *rpsE* gene (encoding ribosomal protein S5),^[^ ^33^, ^34^ ^]^ which was resist to rifampicin, streptomycin, and spectinomycin in *E. coli*, respectively. CTRLE based on the MS2‐MCP system (T5DNAP4M‐MS2 and T7DNAP3M‐MS2), each individually targeting to the aforementioned three genes, were constructed and transformed into *E. coli* TG1 (**Figure** **4**a). The mutation frequency was subsequently evaluated according to the ratio of antibiotic‐resistant colonies to the total number of colonies. Compared to the wild‐type strain, strains harboring the developed mutator displayed a significantly elevated mutation frequency, ranging from approximately 347 to 98 000‐fold higher (Figure 4b–d). T5DNAP4M‐MS2 targeting *rpoB* or *rpsL* exhibited a slightly lower mutation frequency compared to T7DNAP3M‐MS2, while it showed a slightly higher frequency when targeting *rpsE*, which may be influenced by the mutation spectra difference between T5DNAP4M and T7DNAP3M. Furthermore, MS2‐mediated recruitment of T5DNAP4M and T7DNAP3M did contribute to significantly reduced off‐target mutation frequencies, at the 3 locus used above (Figure S5a–c, Supporting Information). T5DNAP4M‐MS2 exhibited 96%, 99.8%, and 95.5% reductions at *rpoB*, *rpsE*, and *rpsL* respectively, whereas the corresponding values for T7DNAP3M‐MS2 were 74.3%, 98%, and 95.5%. T7DNAP3M‐based mutators resulted in over 2‐fold higher off‐target rate than T5DNAP4M regardless of the presence or absence of MS2‐mediated recruitment.

To further investigate the capacity of CTRLE for multiplexed synchronous in situ mutagenesis, three tandem sgRNA cassettes targeting *rpoB*, *rpsL*, and *rpsE* were first designed to evaluate their ability to confer resistance to rifampicin, streptomycin, and spectinomycin, respectively (Figure 4e). As depicted in in Figure 4b–d, the mutation frequencies generated at individual gene locus by T7DNAP3M‐MS2 carrying the three tandem sgRNA cassettes were comparable to those produced by T7DNAP3M‐MS2 carrying a single sgRNA. In contrast, T5DNAP4M‐MS2 carrying the three tandem sgRNA cassettes showed 1–2 orders of magnitude lower mutation frequencies at individual gene loci than that carrying a single sgRNA, potentially attributable to resource competition among multiple sgRNAs for mutator T5DNAP4M‐MS2. These results demonstrated MS2‒MCP‐based CTRLE with tandem sgRNA cassettes had great potential to perform multiplexed synchronous in situ mutagenesis.

Furthermore, T5DNAP4M‐MS2 and T7DNAP3M‐MS2, each equipped with three tandem sgRNA cassettes, were applied into the simultaneous and continuous evolution of *rpoB*, *rpsL*, and *rpsE* for generating resistant strains to three types of antibiotics (Figure 4e). Almost no single colony on plates containing three antibiotics was observed in initial mutagenic cultures obtained by either T5DNAP4M or T7DNAP3M through a round of mutagenesis. Therefore, two cultures were passaged at 1:100 dilutions under the increasing concentrations of three antibiotics ranging from 0 to 200 µg mL^−1^ as independent evolutionary trajectories resulted from T5DNAP4M‐MS2 and T7DNAP3M‐MS2, respectively. For each passage, 4 colonies were picked and the *rpoB*, *rpsL*, and *rpsE* genes were amplified by PCR for sequencing. Three rifampicin‐spectinomycin‐streptomycin resistant strains, M1, M2, and M3 were predominantly enriched in medium containing all three antibiotics after 12 passages in 8 days (**Table** **1**, Figure S6, Supporting Information). These mutations conferred strain significantly increased rifampicin, spectinomycin, and streptomycin tolerance from 0 to 200 µg mL^−1^. Compared with the control strain harboring T5DNAP4M before serial passaging, strains M1, M2, and M3 exhibited significantly enhanced growth on plates containing individual antibiotics (Figure 4f). The V25F and K26N mutations in *rpsE* conferred similar levels of spectinomycin resistance to the host cells (Figure 4f). The strength of rifampicin resistance conferred by mutations in *rpo*B (M1 < M2 < M3) likely determined the advantage of cell growth in medium containing all three antibiotics during evolution campaigns (Figure 4f). These observations may explain why, during CTRLE, strain M1 was outcompeted by M2, and strain M2 was ultimately outcompeted by M3, attributable to the progressively stronger growth advantage of the latter strains (Figure S6, Supporting Information).

**Table 1:** Sequence information of mutant strains rifampicin‐spectinomycin‐streptomycin resistant strains.

Mutant strain	Amino acid changes, nucleotide changes, and base number between the mutation and the nick in rpoB	Amino acid changes, nucleotide changes, and base number between the mutation and the nick in rpsE	Amino acid changes, nucleotide changes, and base number between the mutation and the nick in rpsL
M1	S508P (TCC→CCC), 263 bp; L511R (CTG→CGG), 253 bp	V25F(GTT→TTT), 10 bp	K88R(AAA→AGA), 31 bp
M2	H526L (CAC→CTC), 209 bp	K26N(AAA→AAC), 15 bp	K88R(AAA→AGA), 31 bp
M3	D516N (GAC→AAC), 239 bp	V25F(GTT→TTT), 10 bp	K88R(AAA→AGA), 31 bp

### 2.6 CTRLE Promotes Tat Pathway Evolution for Improved Periplasmic Protein Production

The twin‐arginine translocation (Tat) pathway for protein transport was selected for in situ continuous evolution in *E. coli* to assess the effectiveness of CTRLE for evolving the long pathway.

**Figure 5:**
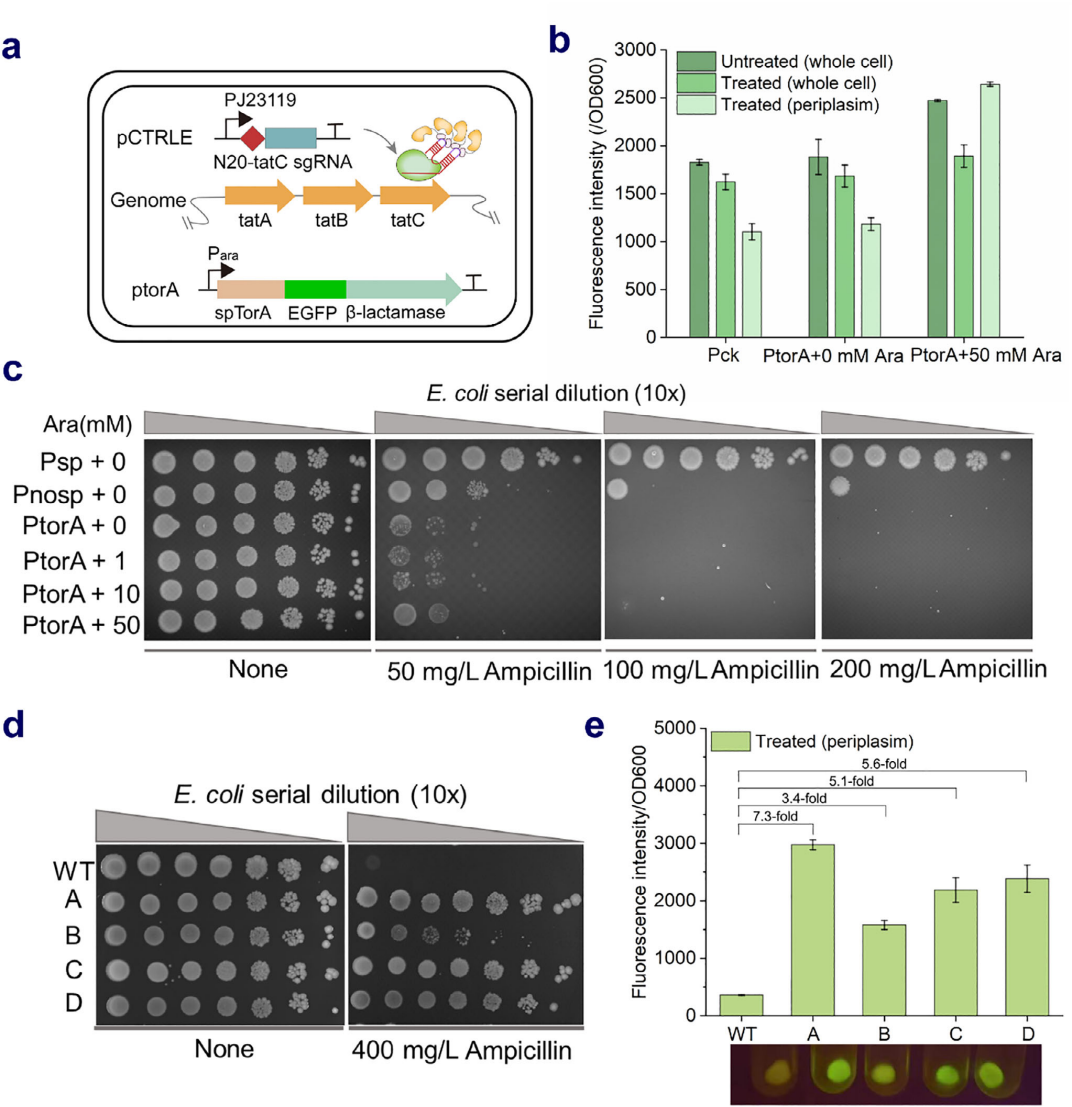
CTRLE for accelerated evolution of the Tat Pathway. a) Schematic diagram of the mutator targeted to the genomic TatC gene and PtorA reporter plasmid. b) Fluorescence assay of whole cell and periplasm for PtorA strain upon addition of 0 and 50 mm arabinose (Ara) before and after using the arginine method (*n* = 3, Data are presented as mean ± SD). c) Cell Growth of strains carrying the Psp, Pnosp, or PtorA plasmid on agar plates containing different concentrations of ampicillin. Psp plasmid contained a constitutively expressed β‐lactamase gene with an N‐terminal signal peptide targeting the general secretory (Sec) pathway. The Sec‐dependent signal peptide was removed from the Psp plasmid, resulting in the Pnosp plasmid. d) Analysis of different evolved mutants A, B, C, and D carrying the mutator and ptorA plasmid through serial passage, taking strain at the beginning of the passage as control (WT). e) Periplasmic EGFP fluorescence from enriched mutants (*n* = 4, Data are presented as mean ± SD).

The Tat translocase consisting of three membrane proteins (TatA, TatB, and TatC), is a route for delivering fully folded native and heterologous proteins into the periplasmic space of *E. coli*. It has been proved that enhancing the translocation ability of the Tat system helps to increase the production of heterologous proteins exported to the periplasm.^[^ ^14^, ^35^ ^]^ Since T5DNAP4M‐MS2 demonstrated lower off‐target effects for both plasmid and genomic DNA compared to T7DNAP3M‐MS2, T5DNAP4M‐MS2 with a single sgRNA targeting the 3′ end of the genomic *tat*C gene was designed to generate efficient translocase variants (**Figure** **5**a). PtorA was constructed as a reporter plasmid, which contained a fusion gene cassette including the encoding sequence of TorA signal peptide recognized by the Tat translocation system, EGFP, and β‐lactamase downstream of promoter P_ara_ (Figure 5a). Upon induction with 50 mm arabinose, the strain harboring PtorA showed an approximately onefold increase in periplasmic fluorescence intensity compared to the non‐induced control (Figure 5b). Meanwhile, the PtorA strain exhibited inhibited growth on plates containing 100 µg mL^−1^ ampicillin (Figure 5c). This method allowed for dual screening of the TatABC mutant library by combining periplasmic fluorescence detection with ampicillin resistance selection.

Mutator T5DNAP4M‐MS2 targeting *tat*C was transformed with plasmid PtorA into *E. coli* TG1. Four replicates were passaged at 1:100 dilutions with increasing concentrations of ampicillin. At the end of the evolution campaigns, 8 colonies were isolated from each culture for sequencing analysis. It was found that all the 8 colonies from each culture carried the mutations in tatABC, resulting in a total of 4 different TatC mutations localized within 183 bp from the nick (**Table** **2**). Through further extensive sequencing of mutant libraries, six mutations (EvoL1–6) were identified in either the *tatA* or *tatB* genes, with detectable mutations occurring as far as 1531 bp from the nick site (Figure S8, Table S4, Supporting Information). These findings are generally consistent with our previous result that the mutation window of the CTRLE system spans approximately 2000 bp. 4 TatC variants showed significantly enhanced viability compared to the wild‐type strain in the presence of 400 µg mL^−1^ ampicillin (Figure 5d). Further analysis by testing fluorescence level in periplasm showed that 4 strains exhibited over threefold higher fluorescence intensity than the wild‐type strain (Figure 5e). Strains A, C, and D displayed stronger periplasmic fluorescence intensity compared to strain B, a trend concordant with their superior ampicillin resistance profile. The correlation between periplasmic fluorescence and ampicillin resistance may reflect improved protein translocation efficiency of the Tat system in these mutants.

**Table 2:** Sequence information of mutant strains (Replicate indicates the number of evolutionary cultures with the same mutation within the tatABC genes).

Mutant strain	Amino acid changes	Nucleotide changes	Base number between the mutation and the nick	Replicate
A	TatC(E244G)	GAA→GGA	1 bp	4
B	TatC(N242T)	AAT→ACT	6 bp	1
C	TatC(E244insertion)	GAA→GGAA	1 bp	1
D	TatC(I183S, E244G)	183:ATT→AGT, 244:GAA→GGA	183 bp	2

### 2.7 Universal and Efficient Application of CTRLE in *B. subtilis* and *K. lactis*

**Figure 6:**
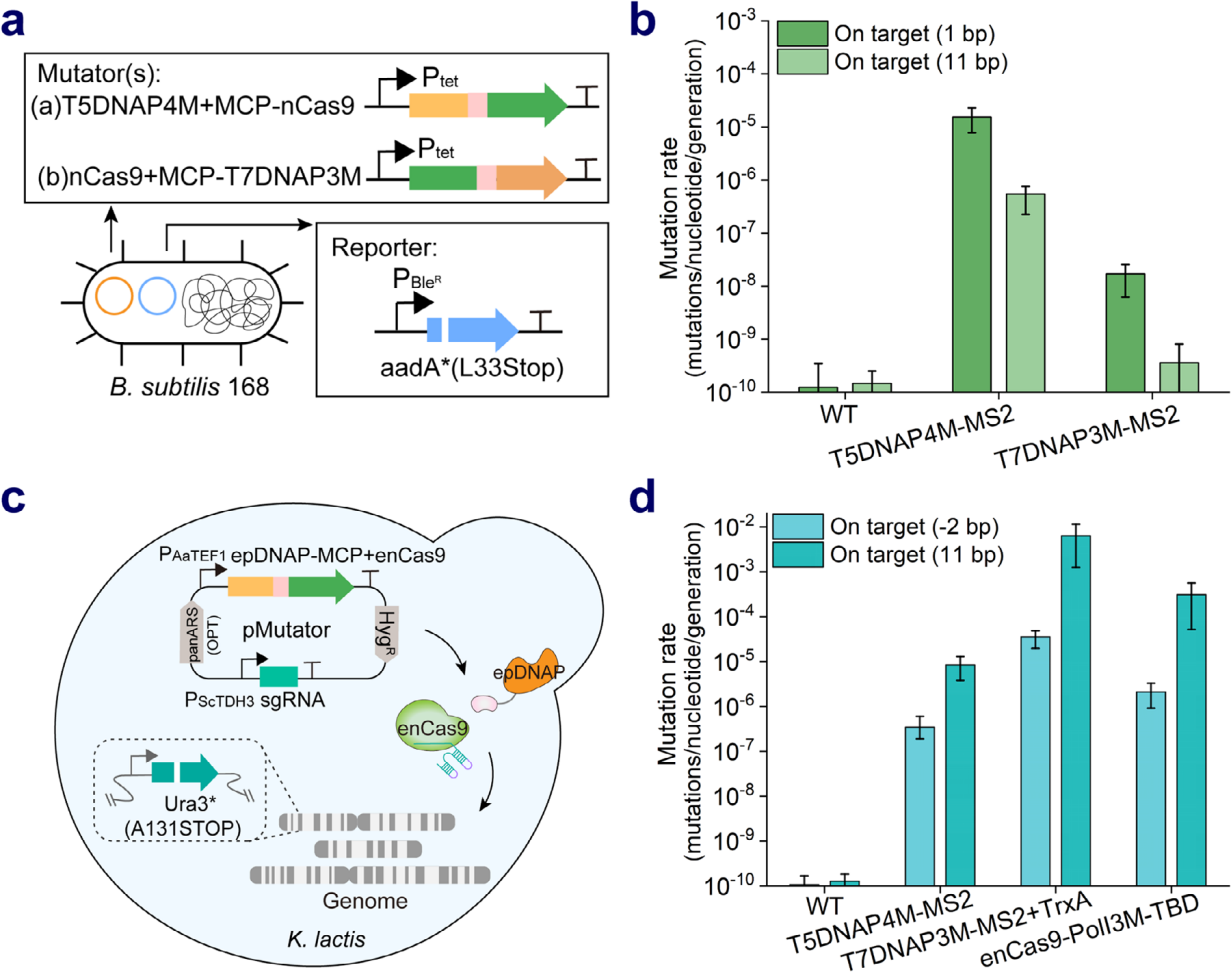
Universality of CTRLE in *B. subtilis* and *K. lactis*. a) Mutator and reporter plasmids were constructed and transformed into *B. subtilis* 168. Mutator plasmid encoded T5DNAP4M or T7DNAP3M fusion with MCP, nCas9, and MS2 aptamer‐modified sgRNA. Gene aadA* (aadA L33STOP) taken as a reporter for measuring mutation rate was expressed from another plasmid. b) The mutation rates generated by T5DNAP4M or T7DNAP3M mutator in *B. subtilis* 168 (*n* = 4, Data are presented as mean ± 95% confidence intervals). c) *K. lactis* GG799 strain that genomically encoded Ura3* (Ura3 A131STOP) was constructed and employed as a uracil auxotrophic selection marker to evaluate the reversion assay. Mutator plasmid contained the coding sequence of T5DNAP4M or T7DNAP3M fusion with MCP, enCas9 controlled by the constitutive AaTEF1 promoter (PAa_TEF1_), and MS2 aptamer‐modified sgRNA targeting Ura3* gene. d) The mutation rates generated by T5DNAP4M mutator, T7DNAP3M mutator with TrxA alone, and EvolvR (enCas9‐PolI3M‐TBD) in *K. lactis* (*n* = 4, Data are presented as mean ± 95% confidence intervals). Two nucleotides from 5′ of the nick site were defined as position −2 bp.

CTRLE exhibited a high mutation rate and an expanded mutation window in *E. coli*. To investigate whether it could applied in other prokaryotic cells, *B. subtilis*, a model bacterium that widely used in the production of industrial enzymes and biotechnology research,^[^ ^36^ ^]^ was selected as host. The gene *aadA* containing the TAA premature nonsense codon was first evaluated in *B. subtilis* 168 (Figure S7a, Supporting Information). Although the sensitivity of strain to spectinomycin was lower than that of *E. coli*, 100 µg mL^−1^ spectinomycin was sufficient for revertant mutation screening (Figure S7b, Supporting Information). Then the aadA* gene was substituted for the *ble* gene that was located in the pP43NMK plasmid containing the sgRNA cassette, producing the reporter plasmid. The low‐cpoy plasmid pHT01 expressing T5DNAP4M or T7DNAP3M mutator was transformed into *B. subtilis* harboring a reporter plasmid to estimate mutation rates (Figure S7c, Supporting Information). It was found that the mutation rate of T5DNAP4M mutator was elevated by 1.2 × 10⁵‐fold (1 bp) and 3.7 × 10^3^‐fold (11 bp) from the nick site compared to the wild‐type strain, which was slightly lower than the results in *E. coli* (**Figure** **6**b). Notably, T7DNAP3M only exhibited a 137‐fold and 2.7‐fold higher than wild‐type strain at these respective positions (Figure 6b), which may be due to the lack of expression of processive factor thioredoxin (TrxA) from *E. coli* for T7DNAP3M.

Subsequently, CTRLE was also established in yeast *K. lactis* which is generally regarded as a safe (GRAS) microorganism and has great potential for the production of food‐grade and therapeutic proteins.^[^ ^37^ ^]^ Gene Ura3* (Ura3 A131STOP) containing a premature termination codon TAA was integrated into the *K. lactis* GG799 genome. The coding sequences of T5DNAP4M or T7DNAP3M mutator were codon‐optimized for yeast, and placed downstream of the constitutive AaTEF1 promoter using pUDP002 as a vector to construct the mutator plasmid. Particularly, the codon‐optimized *trxA* gene from *E. coli* was co‐expressed with T7DNAP3M mutator (Figure 6c). The results showed that the maximum mutation rates of T5DNAP4M and T7DNAP3M mutators were 6.6 × 10⁴‐fold and 5 × 10⁷‐fold higher than the wild‐type strain, respectively (Figure 6d). Interestingly, T7DNAP3M mutator exhibited a mutation rate approximately 20‐fold higher than EvolvR (enCas9‐PolI3M‐TBD).^[^ ^16^ ^]^ In addition, due to the strict PAM sequence requirement of the CRISPR system, mutation rates at 2 bp from the 5′ end of the nick, which is close to 1 bp from the 3′ end of the nick, were evaluated. The mutation rates of all 3 mutators proximal to the 5′ end of the nick were one to two orders of magnitude lower than those near the 3′ end (Figure 6d), which is consistent with previous reports of low‐frequency mutations occurring 5′ of the nick site.^[^ ^16^ ^]^ Altogether, these results demonstrated that CTRLE was a universal and effective system in different industrial microorganisms.

## 3 Discussion

Laboratory evolution is a powerful approach for customizing enzymes or microbial cells with desired functions. The two cores of this technology involve in vivo mutagenesis and a screening strategy.^[^ ^38^, ^39^ ^]^ In vivo mutagenesis can autonomously generate mutations dependent on endogenous or heterologous mutagenic factors. The commonly used in vivo mutagenesis methods randomly mutate the entire genome by spontaneous mutation, physical or chemical mutagenesis, which is time‐consuming and may affect host fitness and screening for beneficial mutations. To address this issue, researchers have developed various in vivo targeted mutagenesis tools enabling localized hypermutation at targeted loci exceeding the host background (**Table** **3**). Targeted mutagenesis of genomic loci has recently attracted increasing attention from researchers due to its ability to explore the optimized function of protein complexes and microbial chassis cells. CRISPR‐guided hypermutation systems, popular examples such as CRISPR‐X^[^ ^27^ ^]^ and EvolvR,^[^ ^16^ ^]^ enable in situ mutagenesis of user‐defined loci with programmable targeting (Table 3). CRISPR‐based base editors have extended mutation windows that can reach dozens of bases or even 55 kb and enable multiple types of base transitions and conversions by fusing different endonucleases with deaminases.^[^ ^13^, ^14^, ^40^ ^]^ Nevertheless, their utility is constrained by biased mutational spectra, covering only a subset of all possible base substitutions. EvolvR can achieve all types of substitutions utilizing error‐prone DNAP, but is limited by a narrowed mutation window length (within 350 bp). In addition, the recently developed T7ACE hypermutation system^[^ ^11^ ^]^ employs error‐prone T7 RNA polymerase to synthesize single‐stranded DNA that can anneal to the lagging strand during genome replication, and introduces all substitutions over lengths of 1 kb at the targeted locus. Thus, it is urgent to develop controllable, targeted hypermutation tools with unrestricted mutation spectra and expanded mutagenesis windows for covering long genes or entire metabolic pathways in microbial cells.

**Table 3:** Performance metrics of current in vivo targeted mutagenesis tools.

Name	Mutagenic factor	Mutation rate [mutations per bp per generation]	Mutagenesis region and window length	Validated hosts
PACE^[^ ^2^ ^]^	MP6 (DNAQ926, dam, seqA, emrR, ugi, and cda1)	6.2 × 10^−6^	M13 phage genome, global mutagenesis	*E. coli*
OrthoRep^[^ ^3^ ^]^ BacRep^[^ ^4^ ^]^ or EcORep^[^ ^5^ ^]^	A highly error‐prone orthogonal DNAP‐DNA plasmid pair	~1 × 10^−5^;^[^ ^3^ ^]^ 6.8 × 10^−7^;^[^ ^4^ ^]^ 9.13 × 10^−7^.^[^ ^5^ ^]^	Whole liner plasmid, several kilobases	*S. cerevisiae*, *B. thuringiensis*, *E. coli*.
TADR^[^ ^6^ ^]^ or T7‐ORACLE^[^ ^7^ ^]^	Low‐fidelity DNA replisome	2.3 × 10^5^‐fold on‐target; 1.73 × 10^−5^	Whole circular plasmid, several kilobases	*E. coli*
CRISPR‐based base editors^[^ ^13^, ^14^, ^40^ ^]^	Base deaminases recruited by the Cas complex	>1 × 10^−3^ in hotspots; biased mutation type.	partial region in the genome, generally <100 bp	Mammalian cells, bacteria, yeast.
EvolvR^[^ ^16^ ^]^ or OMEGA‐R^[^ ^41^ ^]^	Error‐prone DNAP recruited by Cas complex	7 770 000‐fold on‐target;^[^ ^16^ ^]^ 350 000‐fold on‐target;^[^ ^41^ ^]^ dozens of fold off‐target.	partial region in plasmid or genome, <350 bp	*E. coli*, *S. cerevisiae*, *B. subtilis*.
T7ACE^[^ ^11^ ^]^	Error‐prone T7 RNAP mutant synthesizing single‐stranded DNA	2800‐fold on‐target	partial region in the genome, ~1000 bp	*E. coli*, *S. cerevisiae*.
CTRLE	Error‐prone DNAP recruited by Cas complex	1 100 000‐fold on‐target, ~43‐fold off‐target	partial region in plasmid or genome, ~2000 bp	*E. coli*, *B. subtilis*, *K. lactis*.

Previous studies have shown that utilizing the increased processivity of PolI3M fused to nCas9, EvolvR results in an improved mutation window length, while more processive Phi29 DNAP from family B of DNAPs did not increase the mutation window.^[^ ^16^ ^]^ The processivity of DNAPs would be a key factor affecting the lengths of error‐prone strand synthesis from the nick site generated by nCas9. Therefore, the polymerases with higher DNA processivity from bacteriophage were considered as alternatives. T5 and T7 DNAPs, which are phage replicative DNAPs with respective molecular weights of approximately 97.6 and 79.5 kDa, are slightly smaller than PolI with a molecular weight of approximately 103.1 kDa, belong to family A of DNAPs as well as PolI, and share extensive sequence homology with PolI.^[^ ^24^, ^42^, ^43^ ^]^ T5 DNAP is highly processive and can perform strand‐displacement DNA synthesis similar to PolI from nicked duplex DNA without other auxiliary proteins. T7 DNAP likewise possesses high processivity in the presence of *E. coli* thioredoxin,^[^ ^44^ ^]^ and carries out limited strand displacement synthesis at nicked DNA, which could be further catalyzed by T7 gene 4 protein and gene 2.5 protein.^[^ ^45^, ^46^, ^47^ ^]^ While T4 DNAP belonging to family B of DNAPs is low processive and cannot catalyze strand displacement synthesis from the nicked site in the absence of multiple T4 accessory proteins.^[^ ^21^, ^48^, ^49^ ^]^

Here, T5 and T7 DNAP‐based mutators were constructed by fusion of mutagenic T5 and T7 DNAP mutants with nCas9, and successfully resulted in target mutations. Interestingly, two error‐prone T5 DNAP mutant fusions, T5DNAP3M‐ and T5DNAP4M‐nCas9, exhibited mutation rates 1.07 × 10^5^‐fold and 7.08 × 10^5^‐fold higher than the wild‐type strain at 1 bp from the nick. T5DNAP4M‐nCas9 and nCas9‐T7DNAP3M generated mutation rates of exceeding 1.8 × 10^−7^ per base per generation at 347 bp from nick, which is approximately 20‐fold higher than EvolvR (enCas9–PolI3M–TBD).^[^ ^16^ ^]^ When nCas9 was replaced with enCas9, T5DNAP4M‐enCas9 and enCas9‐T7DNAP3M maintained the detectable mutation rates approximately 10^−7^ per base per generation (1000‐fold of wild‐type *E.coli*) at 2 kb from the nick site, which indicating these systems can efficiently achieve all types of substitution mutations within a 2‐kb targeted region.

However, T5DNAP4M‐nCas9 and nCas9‐T7DNAP3M caused considerable off‐target levels. MS2‐mediated recruitment of T5DNAP4M and T7DNAP3M alleviated the DNAP‐dependent off‐target rate by 96.8% and 54.8%, respectively, while maintaining the original targeted mutation rate. Notably, the off‐target rate of T5DNAP4M recruited by the MS2‐MCP system to nCas9 was comparable to that of EvolvR (enCas9–PolI3M–TBD).^[^ ^16^ ^]^ The SpyCatcher/SpyTag system was also employed for co‐localized expression of nCas9 and epDNAP, and generated significant decreases in targeted mutation rate, although off‐target rate was reduced to within 10‐fold of wild‐type *E. coli*. On the contrary, in another recently reported similar work where nCas9 was replaced with a smaller nickase enIscB, SpyCatcher/SpyTag‐mediated colocalization of enIscB and PolI3M‐TBD exhibited a major increase in targeted mutation rate without affecting off‐target rate.^[^ ^41^ ^]^ The reason for this difference may be that dual SpyTag repeats used in this study led to greater steric hindrance and DNA inaccessibility. These results demonstrated an appropriate approach for spatial separation of nickase and epDNAP contributes to improving the ratio of on‐ and off‐target mutation rate, which may benefit from the increased activity and flexibility of proteins.

Another primary feature of CTRLE is its high inducibility for controlling the mutagenesis process. Previous studies on targeted hypermutation systems focused generally on a high targeted mutation rate, paying less attention to the leaky mutation rate during mutagenesis switch‐off periods. Here, the tetracycline‐inducible promoter P_tet_ had no leakage when expressing EGFP, but resulted in a relatively high targeted mutation rate when expressing mutators in the absence of tetracycline. Introducing the dTnpB‐based transcriptional repression system could mitigate this issue by substantially reducing leakage of mutator, resulting in a more rigorous hypermutation system. Therefore, CTRLE was developed as a properly regulated mutagenesis system by limiting leakage without additionally eliminating the mutator. Furthermore, CTRLE was applied into the continuous evolution of three genomic loci and the Tat translocase pathway in *E. coli*. Indeed, CTRLE also exhibited robust mutagenesis efficiency across divergent species (*E. coli*, *B. subtilis*, and *K. lactis*), underscores its potential as a universal mutagenesis tool for microbial and protein engineering.

Furthermore, antibiotic‐resistant mutants and transporter protein mutants achieved by the CTRLE system hold significant potential for biotechnological research and industrial applications, such as studying strain resistance mechanisms and enhancing industrial production of recombinant proteins. The CTRLE system's capability for multi‐locus targeted mutagenesis and its broad mutation window make it an ideal tool for evolving microbial cell factories with improved performance. It can be used to optimize complex metabolic pathways for the production of high‐value compounds (e.g., natural products, biofuels, pharmaceuticals) by simultaneously engineering multiple enzymes or proteins involved in a pathway. The CTRLE system can also accelerate the discovery of industrial enzyme variants with improved properties such as stability, catalytic activity, and substrate specificity, thereby optimizing various industrial bioprocesses.

Additionally, some limitations of CTRLE could be addressed in future work. First, off‐target mutation rates of T5DNAP4M and T7DNAP3M recruited by the MS2‐MCP system were approximately 43‐fold and 215‐fold higher than that of the wild‐type cell, they are still insufficient and need to be further optimized by utilizing an improved co‐localization strategy and nickase or epDNAP mutants with low off‐target activity for maintaining the integrity of the host genome during continuous evolution for a long timescale. Second, the targeted mutation rate of CTRLE needs to be increased within the expanded mutation window. Introducing corresponding auxiliary proteins for epDNAP would further enhance the activity and processivity of epDNAP. In addition, integrated expression of large‐sized proteins of CTRLE may moderately mitigate cell growth burden, and make it easier to construct plasmids containing designed sgRNA through simple molecular operations.

In conclusion, a targeted hypermutation system CTRLE was established based on the coordination of bacteriophage T5/T7 epDNAP and nickase nCas9, which exhibits an expanded mutagenesis window of up to 2‐kb, low off‐target activity, unrestricted types of substitution mutations, and high controllability. Continuous and rapid evolution of three genomic loci and the Tat translocase pathway further demonstrated the multiplexed in situ mutagenesis ability of CTRLE. These findings suggest that CTRL would be promising for the accelerated evolution of proteins, genetic elements, pathways, and chassis cells.

## 4 Experimental Section

### Plasmids and Strain Cultivation

The strains, plasmids, and protospacer sequences used in this study can be found in Tables S1 and S2 (Supporting Information). All DNA fragments were PCR‐amplified using primers listed in Table S3 (Supporting Information). Plasmid construction was performed using DNA fragments and the seamless cloning kit obtained from Sangon Biotech (Shanghai, China). For gene cloning, strain *E. coli* JM109 was cultivated at 37 °C in Luria–Bertani (LB) broth. For in vivo mutagenesis, *E. coli* TG1, *E. coli* MG1655, *E. coli* W3110, and *B. subtilis* 168 were cultivated at 37 °C in LB medium, except *K. lactis* was cultivated at 30 °C in Yeast peptone dextrose (YPD) medium. Unless otherwise specified, the final concentrations of antibiotics added were usually as follows: 30 µg mL^−1^ chloromycetin, 50 µg mL^−1^ kanamycin, 50 µg mL^−1^ spectinomycin, 50 µg mL^−1^ rifampicin, 50 µg mL^−1^ streptomycin, 100 µg mL^−1^ ampicillin, and 250 µg mL^−1^ Hygromycin B.

### Fluctuation Analysis and Mutation Rate Calculation

The mutator plasmid and target plasmid (pTarget) were cotransformed into *E. coli* TG1, which was transferred at a 1: 500 dilution into fresh LB medium containing chloromycetin, kanamycin, and 50 ng mL^−1^ anhydrous tetracycline after recovery culture for 1.5 h. After 14 h for cultivation, diluted cells were coated on LB plates containing chloramphenicol and kanamycin, in the presence/absence 50 µg mL^−1^ of spectinomycin.

For *B. subtilis* 168, the BS‐T5DNAP4M‐MS2 or BS‐T7DNAP4M‐MS2 plasmid was transformed into competent cells harboring the BS‐pTarget‐aadA* plasmid. After recovery for 4 h at 37 °C, 100 µL transformation mix was transferred into fresh 10 mL LB medium containing 6 µg mL^−1^ chloromycetin, 50 µg mL^−1^ kanamycin, and 25 ng mL^−1^ anhydrous tetracycline. Subsequently, cultures were grown for 20 h at 37 °C and dilutions were coated on LB plates containing 6 µg mL^−1^ chloramphenicol and 50 µg mL^−1^ kanamycin, in the presence/absence of 100 µg mL^−1^ spectinomycin.

For *K. lactis* GG799*, 500 ng mutator plasmid was electroporated into competent cells according to the previously reported method.^[^ ^50^ ^]^ After incubation for 1 h at 30 °C, 1 mL transformation culture was transferred into fresh 25 mL YPD medium containing Hygromycin B and then grown to saturation at 30 °C. Cells were harvested, washed, and diluted with sterile water, then plated onto SD plates lacking or adding uracil.

Next, the reversion and total colonies were counted, and the mutation rate was estimated using the online tool Falcor based on Ma–Sandri–Sarkar maximum likelihood method.^[^ ^51^ ^]^ For gain‐of‐function mutation in *aadA* or *Ura3*, 7 missense mutations among 9 base‐pair substitutions were permissive in the stop codon (TAA).^[^ ^17^ ^]^ The resulted mutation rate was multiplied by 9/7, then divided by 3 (base number) and the targeted plasmid copy number, generating the final mutation rate per generation per base.

### Sample Preparation and High‐Throughput Sequencing

The mutator plasmid (T5DNAP4M‐nCas9 or nCas9‐T7DNAP3M) and target plasmid (pTarget) were co‐transformed into *E. coli* TG1. After recovery for 1.5 h at 37 °C, transformation cultures were first inoculated at 1:500 dilution into fresh LB media supplemented with chloromycetin and kanamycin and 50 ng mL^−1^ anhydrous tetracycline, and grown in a 37 °C shaker for 14 h, which was performed 3 parallel cultures for each mutator. Mutagenesis cultures were then passaged at 1:1000 dilutions into fresh LB medium containing antibiotics and induces for 14 h of cultivation. After 19 passages for accumulated mutagenesis, 3 mL sample was harvested from each culture and used for plasmid extraction using a SanPrep Column Plasmid Mini‐Preps Kit (Sangon Biotech). Plasmids obtained from samples were used as templates for generating amplicons of the targeted plasmid, taken pre‐evolutionary target plasmid as a control. PCR products were further purified using the SanPrep Column DNA Gel Extraction Kit (Sangon Biotech). Amplicons were analyzed through library construction, validation, and sequencing using the Illumina NovaSeq platform (GENEWIZ).

### dTnpB‐Based Transcriptional Repression

For expression analysis of T5DNAP4M or T7DNAP3M mutator controlled by Ptet, Pt5test/Pt7test plasmid was transformed with pTarget plasmid into *E. coli* TG1. After recovery for 1.5 h at 37 °C, cultures first inoculated at a 1: 500 dilution into three parallel shake flasks containing chloromycetin and kanamycin, in the absence and presence of anhydrous tetracycline (50 ng mL^−1^ for Pt5test and 75 ng mL^−1^ for Pt7test). After grown for 6 h, cells were obtained, washed, and resuspended in PBS buffer for fluorescence intensity assay. OD600 and EGFP fluorescence (excitation at 488 nm and emission at 520 nm) were measured using a microplate reader (BioTek).

For analysis of dTnpB‐mediated expression regulation, the dTnpB cassette (controlled by Para) was cloned with/without its gRNA cassette targeting to Ptet into Pt5test/Pt7test plasmid to assemble plasmid Pt5test/Pt7test‐dTnpB(‐/+gRNA). The resulting plasmid was transformed with the pTarget plasmid into *E. coli* TG1. Similar to the above procedure, the transformation mix was inoculated at a 1: 500 dilution into fresh LB media containing antibiotics, in the absence and presence of 1 mm arabinose. Fluorescence intensity assay was conducted after 12 h of induction.

### Multiplexed In Situ Mutagenesis and Screening Of Multi‐Functional Strains

For generating antibiotic‐resistant strains, CTRLE harboring nCas9 with sgRNA targeting to rpoB, rpsE, or rpsL were constructed and transformed into *E. coli* TG1. After recovery for 1.5 h at 37 °C, cultures were inoculated at 1:500 dilution into fresh LB media containing kanamycin and anhydrous tetracycline (100 ng mL^−1^ for T5DNAP4M‐MS2 and 75 ng mL^−1^ for T7DNAP3M‐MS2). After grown for 14 h at 37 °C, an appropriate concentration of cultures was coated on LB media containing kanamycin with/without corresponding antibiotic (rifampicin, spectinomycin, or streptomycin). The corresponding resistant frequencies were calculated by counting the number of corresponding resistant colonies and total colonies.

For continuous evolution of antibiotic‐resistant strains, T5DNAP4M‐MS2 or T7DNAP3M‐MS2 with three tandem sgRNA cassettes targeting to rpoB, rpsL, and rpsE was constructed and transformed into *E. coli* TG1. After recovery for 1.5 h at 37 °C, cultures were inoculated at 1:500 dilution into 25 mL of fresh LB medium supplemented with kanamycin and anhydrous tetracycline. Cultures (250 µL) from the previous round of mutagenesis were transferred into 25 mL LB medium. After three rounds of mutagenesis, rifampicin, spectinomycin, and streptomycin were supplemented as selection pressure for mutagenesis and enrichment. The added concentrations of the above three antibiotics were the same and sequentially increased with each subsequent passage (5→10→25→50→75→100→125→150→200 µg mL^−1^). Until OD600 exceeded 2 at 37 °C, the cultures were then passaged for the next round of mutagenesis and enrichment. Two replicates with T5DNAP4M‐MS2 and 2 replicates with T7DNAP3M‐MS2 were performed. After completing the continuous evolution experiment, the stored cultures from a certain round of passages were coated on LB agar plates containing kanamycin, 1mM arabinose, and corresponding concentrations of the three antibiotics for switching from a mutagenic mode to a fidelity state. The resultant single colonies were sequenced for colony PCR products of rpoB, rpsE, and rpsL, respectively.

### Continuous Evolution and Screening of TatABC Pathway Variants

T5DNAP4M‐MS2 with a sgRNA cassette targeting to TatC was constructed and transformed with the PtorA plasmid into *E. coli* TG1. Four replicates were performed for the continuous evolution experiment. After one round of mutagenesis, 8 µL cultures were transferred into a 96‐deep well plate containing 800 µL LB medium supplemented with chloromycetin, kanamycin, anhydrous tetracycline, and varying concentrations of ampicillin. Ampicillin concentration gradient selection was performed by increasing the antibiotic pressure with passage cycles (75→100→150→200→400 µg mL^−1^). When OD600 reached over 1, the cultures were diluted 1:100 in 800 µL fresh LB medium and grown at a 37 °C shaker (220 rpm) for the next round of evolution. After the final selection, cells through 8 consecutive passages were plated on LB media with chloromycetin, kanamycin, 1 mm arabinose, and 400 µg mL^−1^ ampicillin, and single colonies were performed colony PCR for Sanger sequencing of the Tat ABC gene. Sorted variants were then validated via growth rate and periplasmic EGFP assay.

### Periplasmic EGFP Assay

Cells were harvested by centrifugation and washed once with PBS buffer at 4 °C. Next, cells were resuspended at a dilution of 1:10 with arginine solution (0.4 m, pH 8.0) and incubated for 45 min at 4 °C. Then, the supernatant was extracted from the mixed solution by centrifugation at 5000 rpm for 10 min, and subsequently transferred directly into a microplate for periplasmic fluorescence detection.

### Statistical Analysis

At least three biological replicates were conducted to determine statistical values (means ± confidence intervals or SD). OriginPro (version 95C) was used for graphing and statistical analyses. Two‐sided student's *t*‐test was utilized to assess differences between groups, with statistical significance defined as *P < 0.05, **P < 0.01, and ***P < 0.001.

## Conflict of Interest

The authors declare no conflict of interest.

## Author Contributions

S.C. and G.Z. designed the experiments. S.C., C.X., P.Y., and L.Q. performed the experiments. S.C. and G.Z. analyzed the data S.C. and G.Z. wrote and revised the manuscript, and J.Z., J.L., G.D., G.Z., and C.J. supervised the project. All the authors read and approved the final manuscript.

## Supporting information



Supporting Information

## Data Availability

The data that support the findings of this study are available from the corresponding author upon reasonable request.
