# Supplementary material for: CRISPR‐DNA Polymerase Assisted Targeted Mutagenesis for Regulable Laboratory Evolution
Source: Adv Sci (Weinh). 2025 Sep 23;12(45):e11448. doi: 10.1002/advs.202511448 (PMC12677585; doi:10.1002/advs.202511448)
Supplement: Supplementary file 1 — Supporting Information [file ADVS-12-e11448-s001.docx]

**Supporting Information**

***CRISPR-DNA Polymerase Assisted Targeted Mutagenesis for Regulable Laboratory Evolution***

Shuaili Chen^1,2^, Xiangdi Chen^1,2^, Yifan Peng^1,2^, Qinghua Li^1,2^, Jingwen Zhou^1,2,3^, Jianghua Li^1,2,3^, Guocheng Du^1,2,3^, Jian Chen^1,2,3^, Guoqiang Zhang^1,2,3^*

^1^ Science Center for Future Foods, Jiangnan University, 1800 Lihu Road, Wuxi, Jiangsu 214122, China

^2^ School of Biotechnology and Key Laboratory of Industrial Biotechnology of Ministry of Education, Jiangnan University, Wuxi 214122, China

^3^ Jiangsu Province Basic Research Center for Synthetic Biology, Jiangnan University, Wuxi 214122, China

*Corresponding authors: Guoqiang Zhang

Mailing address: Science Center for Future Foods, Jiangnan University, 1800 Lihu Road, Wuxi, Jiangsu 214122, China

Phone: +86-510-85914371, Fax: +86-510-85914371

E-mail: gqzhang@jiangnan.edu.cn (Zhang GQ)

**Supplementary Figures**


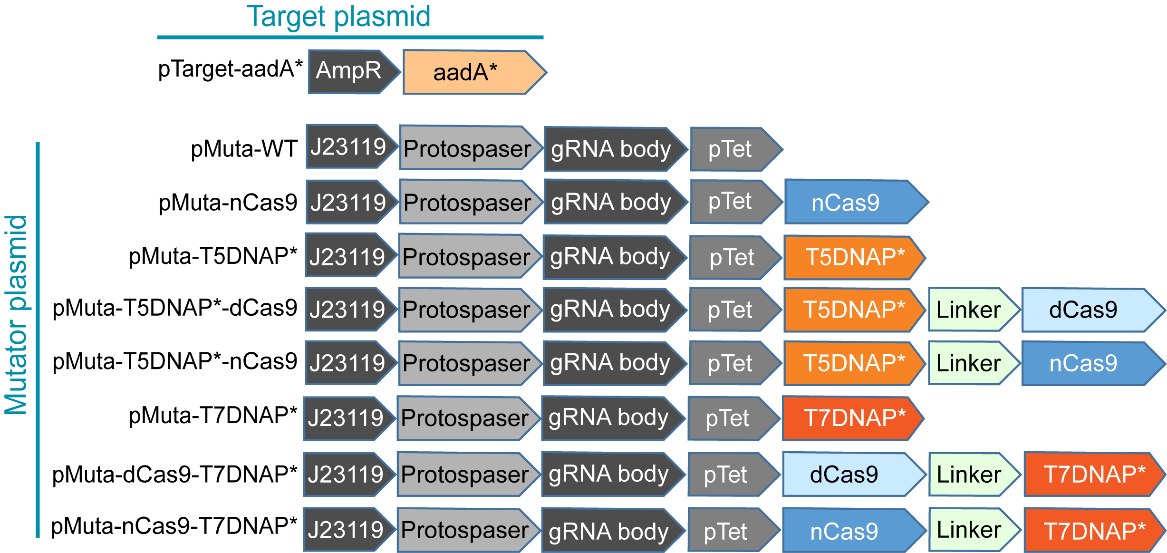


**Figure S1. The structures and designations of mutator and target plasmid.** T5DNAP* represents T5DNAP3M or T5DNAP4M, and T7DNAP* represents T7DNAP3M.

**
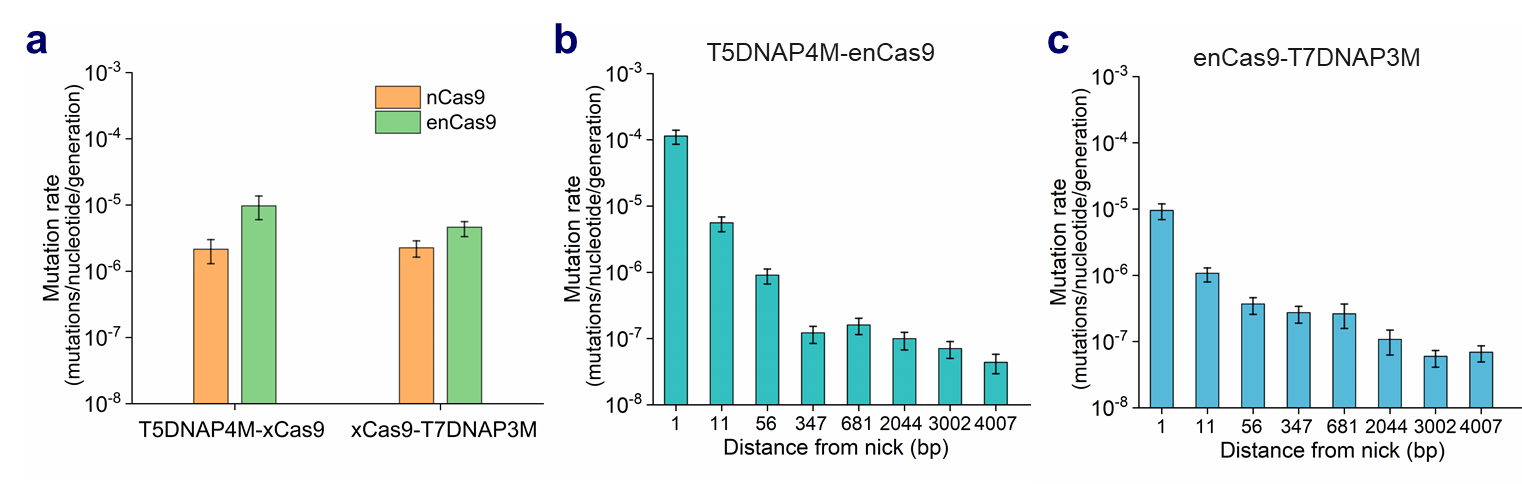
**

**Figure S2. Analysis of mutation rate and mutagenesis window using** **T5DNAP4M or T7DAP3M fused with** **enCas9.** a, Comparison of mutation rate generated by T5DNAP4M or T7DAP3M fused with nCas9 or enCas9. Mutation rates of T5DNAP4M-enCas9 (b) and enCas9-T7DAP3M (c) at different distances from nick.

**
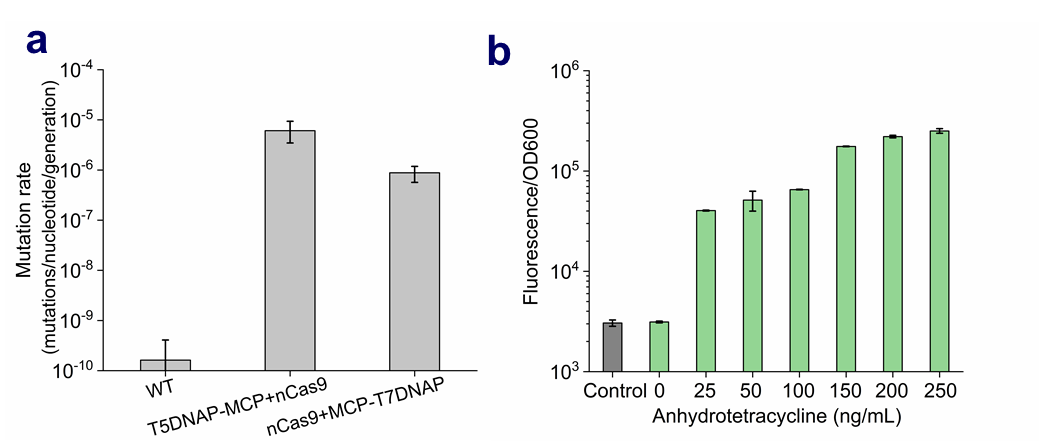
**

**Figure S3. Expression analysis of T5DNAP4M- or T7DNAP3M-based mutator controlled by Ptet promotor.** a, The targeted mutation rate of T5DNAP4M- or T7DNAP3M-based mutator without addition of tetracycline inducer. b, Fluorescence expression of strain harbouring pET-EGFP construct after culturing for 12 hours at a range of tetracycline concentrations.

**
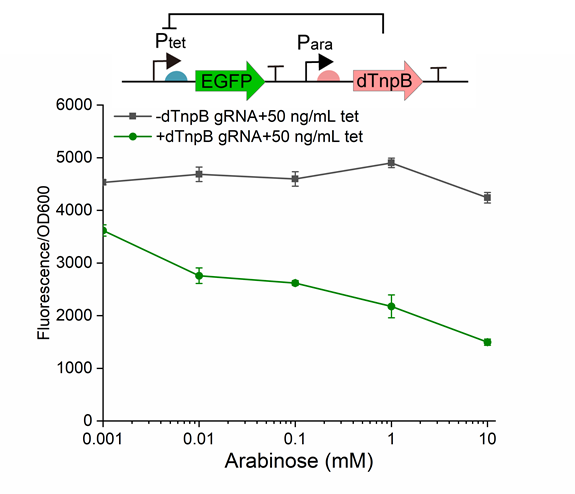
**

**Figure S4.** **Controlling EGFP expression via dTnpB-based transcriptional repression.** Fluorescence intensity were assessed for strains harbouring pET-EGFP-dTnpB where EGFP expression was suppressed via addition of a range of arabinose concentrations, taken strain harbouring pET28a plasmid as control.


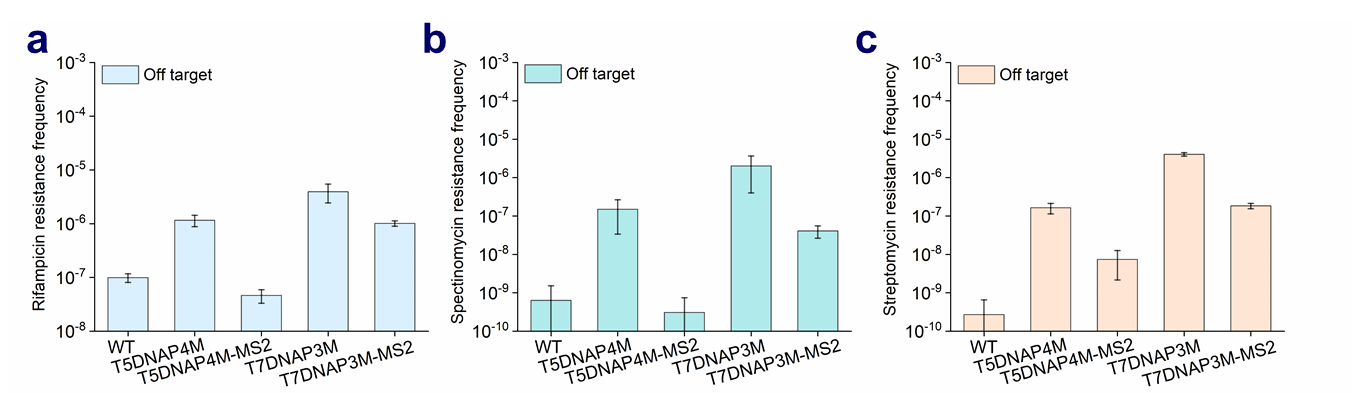


**Figure S5. Off target mutation frequencies generated by CTRL at genomic locus.** Rifampicin (a), spectinomycin (b), and streptomycin (c) resistance frequency produced by CTRL or MS2-mediated CTRL that carried a single sgRNA cassette targeting *dbp*A located in *E. coli* genome.

**
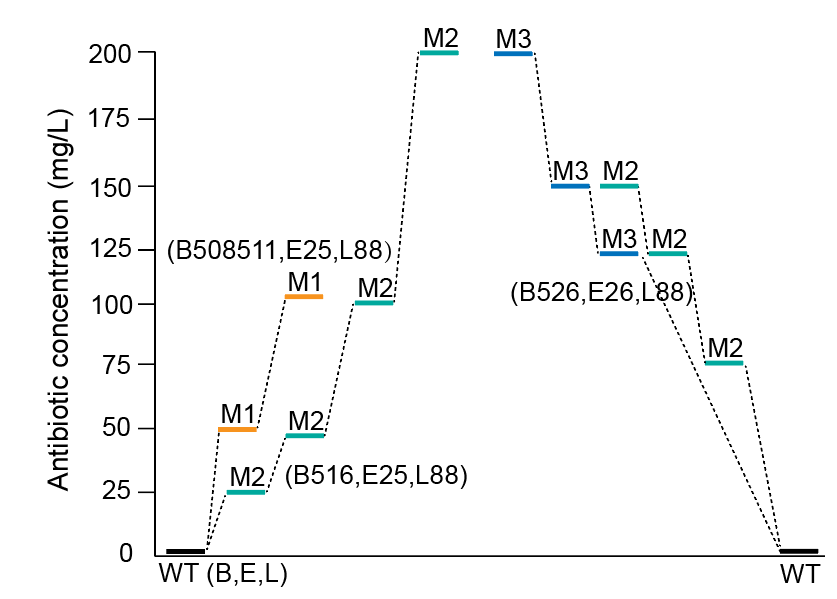
**

**Figure S6. Time course for evolution experiments of RpoB、rpsE和rpsL.** Left and right part represents main mutants enriched in serial passage cultures via continuous evolution mediated by T7DNAP-MS2 and T5DNAP-MS2, respectively. (B, E, L) represents strains bearing origin/evolved rpoB, rpsE, and rpsL, and number in parentheses represents the amino acid where the mutation occurred in corresponding protein.

**
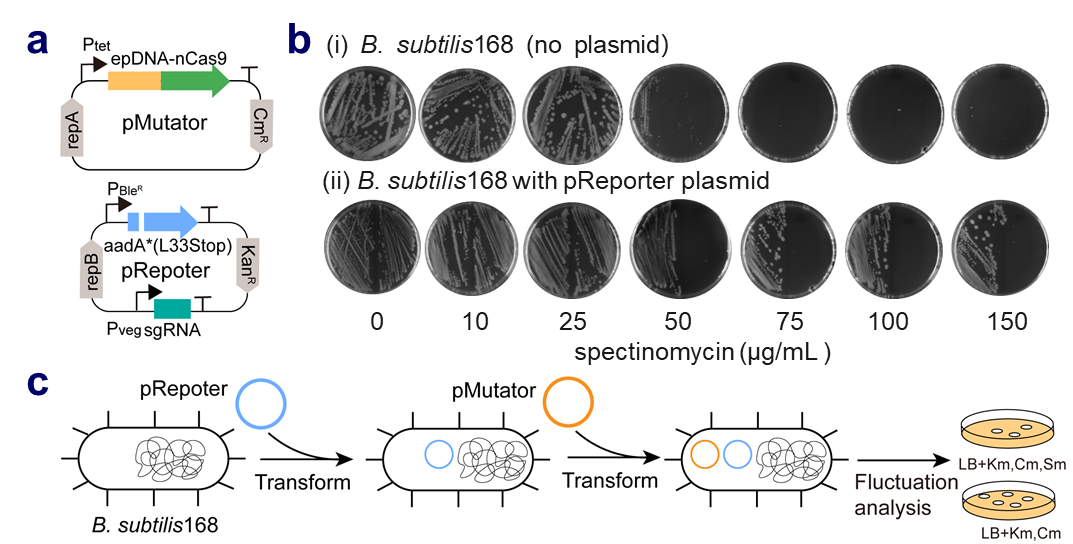
**

**Figure S7. Establishing CTRL in** ***B. subtilis* 168.** a, Schematic diagram of mutator and reporter plasmid for *B. subtilis* 168. b, Growth sensitivity of *B. subtilis* 168 harboring no (i) or reporter plasmid BS-pTarget- aadA or aadA* (ii) to a range of concentrations of spectinomycin. (ii) Left half of plate was coated with strain containing wild type *aadA* gene, the right was coated with strain containing *aadA** (aadA L33Stop) gene. c, The workflow for mutation rate assessment in *B. subtilis* 168.


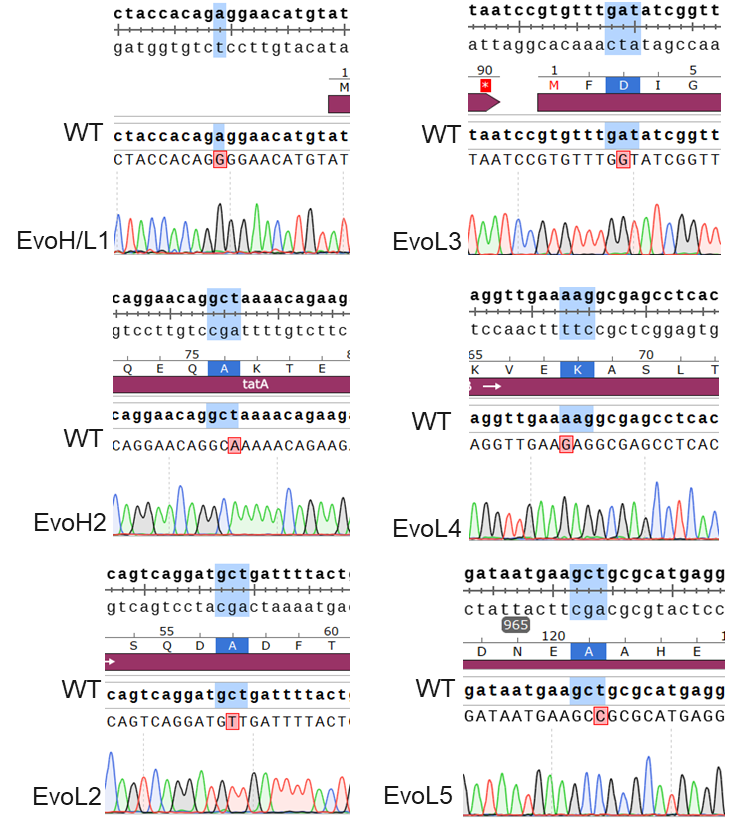


**Figure S8. Identification of mutations in tatA or tatB genes by sequencing.** The mutated bases aligning with origin sequence are framed with red rectangles.

**Supplementary Tables**

**Table S1. strains and plasmids used in this work**

| Strain or Plasmid | Description | Sources |
| --- | --- | --- |
| Strain |  |  |
| *E. coli* JM109 | Wild type for gene cloning | Lab stock |
| *E. coli* TG1 | Wild type for protein expression and in vivo mutagenesis evaluation | Lab stock |
| *E. coli* MG1655 | Wild type for in vivo mutagenesis evaluation | Lab stock |
| *E. coli* W3110 | Wild type for in vivo mutagenesis evaluation | Lab stock |
| *B. subtilis* 168 | Wild type for in vivo mutagenesis evaluation | Lab stock |
| *K. lactis* GG799* | *K. lactis* GG799 carrying Ura3 A131STOP mutation for  in vivo mutagenesis evaluation | This work |
| Plasmid |  |  |
| pACYCDuet-1 | Expression vector, chloramphenicol resistance | Lab stock |
| pET28a (+) | Expression vector, kanamycin resistance | Lab stock |
| pTarget-aadA* | pACYCDuet-1 carrying an inactivated spectinomycin resistance gene *aadA** (aadA L33STOP) cassette | This work |
| pMuta-WT (On or off target) | pET28a carrying a sgRNA cassette targeting to *aadA** gene or *dbpA* and no gene under tetracycline-inducible promoter Ptet | This work |
| pMuta-nCas9 (On or off target) | pET28a carrying a sgRNA cassette targeting to *aadA** gene or *dbpA* and nCas9 (Cas9 D10A) under Ptet promoter | This work |
| pMuta-T5DNAP3M (On or off target) | pET28a carrying a sgRNA cassette targeting to *aadA** gene or *dbpA* and T5DNAP3M (T5DNAP D164A, E166A, and A593R) under Ptet promoter | This work |
| pMuta-T5DNAP4M (On or off target) | pET28a carrying a sgRNA cassette targeting to *aadA** gene or *dbpA* and T5DNAP4M (T5DNAP3M I308V) under Ptet promoter | This work |
| pMuta-T5DNAP3M-dCas9 (On or off target) | pET28a carrying a sgRNA cassette targeting to *aadA** gene or *dbpA* and T5DNAP3M fusion with dCas9 (Cas9 D10A and H840A) via a 23-aa linker under Ptet promoter | This work |
| pMuta-T5DNAP4M-dCas9 (On or off target) | pET28a carrying a sgRNA cassette targeting to *aadA** gene or *dbpA* and T5DNAP4M fusion with dCas9 via a 23-aa linker under Ptet promoter | This work |
| pMuta-T5DNAP3M-nCas9  (On or off target) | pET28a carrying a sgRNA cassette targeting to *aadA** gene or *dbpA* and T5DNAP3M fusion with nCas9 (Cas9 D10A) via a 23-aa linker under Ptet promoter | This work |
| pMuta-T5DNAP4M-nCas9  (On or off target) | pET28a carrying a sgRNA cassette targeting to *aadA** gene or *dbpA* and T5DNAP4M fusion with nCas9 (Cas9 D10A) via a 23-aa linker under Ptet promoter | This work |
| pMuta-T7DNAP3M (On or off target) | pET28a carrying a sgRNA cassette targeting to *aadA** gene or *dbpA* and T7DNAP3M (T7DNAP D5A, E7A, Y64C, F120L, and S399T) under Ptet promoter | This work |
| pMuta-dCas9-T7DNAP3M (On or off target) | pET28a carrying a sgRNA cassette targeting to *aadA** gene or *dbpA* and T7DNAP3M fusion with dCas9 via a 23-aa linker under Ptet promoter | This work |
| pMuta-nCas9-T7DNAP3M (On or off target) | pET28a carrying a sgRNA cassette targeting to *aadA** gene or *dbpA* and T7DNAP3M fusion with nCas9 (Cas9 D10A) via a 23-aa linker under Ptet promoter | This work |
| pMuta-T5DNAP4M-MCP +nCas9 (On or off target) | pET28a containing T5DNAP4M fusion to the N-terminus of MCP (N55K) via a 23-aa linker, followed by intergenic sequence (AAGGAGATATACAT), and downstream *nCas9* gene controlled by Ptet promoter, and carrying a sgRNA scaffold that was modified by two MS2 aptamers (M13) and targeted to *aadA** or *dbpA* | This work |
| pMuta-T5DNAP4M-SPY +nCas9 (On or off target) | pET28a containing T5DNAP4M fusion with a dual SpyTag repeats via a 23-aa linker, followed by intergenic sequence (AAGGAGATATACAT), and downstream nCas9 fusion to the C-terminus of SpyCatcher via a 7-aa linker (GGSGSGLQ) controlled by Ptet promoter, and carrying a sgRNA cassette targeting to *aadA** or *dbpA* | This work |
| pMuta-nCas9+MCP-T7DNAP3M (On or off target) | pET28a containing *nCas9* gene, followed by intergenic sequence (AAGGAGATATACAT), and downstream T7DNAP3M fusion to the C-terminus of MCP (N55K) via a 23-aa linker controlled by Ptet promoter, and carrying a sgRNA scaffold that was modified by two MS2 aptamers (M13) and targeted to *aadA** or *dbpA* | This work |
| pMuta-nCas9+SPY-T7DNAP3M (On or off target) | pET28a containing nCas9 fusion to the N-terminus of dual SpyTag repeats via a 7-aa linker (GGSGSGLQ), followed by intergenic sequence (AAGGAGATATACAT), and downstream T7DNAP3M fusion to the C-terminus of SpyCatcher via a 23-aa linker controlled by Ptet promoter, and carrying a sgRNA cassette targeting to *aadA** or *dbpA* | This work |
| Pt5test | pMuta-T5DNAP4M-MCP+nCas9 plasmid carrying *egfp* gene fused to the C-terminus of nCas9 via a 11-aa linker (GSGGGSGGGGS) | This work |
| Pt5test-dTnpB | Pt5test plasmid carrying dTnpB (TnpB D191A) expressed from arabinose-inducible promoter Para with or without its gRNA targeting to Ptet promoter | This work |
| Pt7test | pMuta-nCas9+MCP-T7DNAP3M plasmid carrying *egfp* gene fused to the C-terminus of nCas9 via a 11-aa linker (GSGGGSGGGGS) | This work |
| Pt7test-dTnpB | Pt7test plasmid carrying dTnpB expressed from Para promoter with or without its gRNA targeting to Ptet promoter | This work |
| T5DNAP4M-MS2-1sg or 3sg | pMuta-T5DNAP4M-MCP+nCas9 plasmid carrying dTnpB expressed from Para promoter with its gRNA targeting to Ptet promoter, and containing MS2-modified sgRNA (nCas9) targeting to *rpoB**/rpsL**/rpsE or rpoB, rpsL,* and *rpsE* | This work |
| T7DNAP3M-MS2-1sg or 3sg | pMuta-nCas9+MCP-T7DNAP3M plasmid carrying dTnpB expressed from Para promoter with its gRNA targeting to Ptet promoter, and containing MS2-modified sgRNA (nCas9) targeting to *rpoB/rpsL/rpsE or rpoB, rpsL,* and *rpsE* | This work |
| pHT01 | *E. coli*-*B. subtilis* shuttle vector, chloromycetin resistance | Lab stock |
| pP43NMK | *E. coli*-*B. subtilis* shuttle vector, kanamycin resistance | Lab stock |
| BS-pTarget-aadA* | pHT01 carrying an inactivated spectinomycin resistance gene *aadA** (aadA L33STOP) and MS2-modified sgRNA expression cassette | This work |
| BS-T5DNAP4M-MS2 | pP43NMK carrying T5DNAP4M-MCP fusion and nCas9 expression cassette | This work |
| BS-T7DNAP3M-MS2 | pP43NMK carrying nCas9 and T7DNAP3M-MCP fusion expression cassette | This work |
| pUDP002 | *E. coli*-*K. lactis* shuttle vector, hygromycin resistance | Addgene |
| EvolvR (enCas9-PolI3M-TBD) | *E. coli*-*Saccharomyces cerevisiae* shuttle vector | Addgene |
| KL-T5DNAP4M-MS2 | pUDP002 carrying T5DNAP4M-MCP fusion, nCas9, and sgRNA expression cassette with | This work |
| KL-T7DNAP3M-MS2 | pUDP002 carrying nCas9, T7DNAP3M-MCP fusion, TrxA and sgRNA expression cassette | This work |
| KL-enCas9-PolI3M-TBD | pUDP002 carrying enCas9-PolI3M-TBD fusion and sgRNA expression cassette | This work |

**Table S2. Protospacer sequences used in this work**

| Name | Sequence (5'-3') |
| --- | --- |
| sgRNA targeting to *aadA** (nicks 1 bp from premature TAA) | ttgctggccgtacattaata |
| sgRNA targeting to *aadA** (nicks 11 bp from premature TAA) | acattaatacggctccgcag |
| sgRNA targeting to *aadA** (nicks 56 bp from premature TAA) | tgatattgatttgctggtta |
| sgRNA targeting to *aadA** (nicks 347 bp from premature TAA) | tgccttggtaggtccagcgg |
| sgRNA targeting to *aadA** (nicks 681 bp from premature TAA) | GGCGAGATCACCAAGGTAGT |
| sgRNA targeting to *aadA** (nicks 2044 bp from premature TAA) | GTCTCATTTTCGCCAAAAGT |
| sgRNA targeting to *aadA** (nicks 3002 bp from premature TAA) | GTTACCTCGGTTCAAAGAGT |
| sgRNA targeting to *aadA** (nicks 4007 bp from premature TAA) | CTTCCACAGCAATGGCATCC |
| sgRNA targeting to dbpA | gcatggaaacagttacaggg |
| dTnpB gRNA targeting to Ptet promoter | agagttattttaccactccc |
| sgRNA targeting to *rpoB* (nicks after nucleotide position 1784) in genome of *E. coli* | ccgtatcgtaaagtgaccga |
| sgRNA targeting to *rpsE* (nicks after nucleotide position 77) in genome of *E. coli* | GTATCTAAAACCGTTAAAGG |
| sgRNA targeting to *rpsL* (nicks after nucleotide position 293) in genome of *E. coli* | GTTCGTTACCACACCGTACG |
| sgRNA targeting to *tatC* (nicks after nucleotide position 730) in genome of *E. coli* | taaagggcgaaatcgggaag |
| sgRNA targeting to *Ura3** (nicks 11 bp from premature TAA) in genome of *K. lactis* | ATTGTTtaaGGCTTGAAACA |

**Table S3. Oligonucleotides used in this study**

| Primer | Template DNA and target products for plasmid construction | Sequence (5'-3') |
| --- | --- | --- |
| aadA*-F | spectinomycin resistance gene aadA with the premature stop codon TAA, gene for constructing pTarget | GCGACTCCTGCATTAGGAAATTTTGTTTATTTTTCTAAATACATTCAAATATGTATCCGCTCATGAG |
| aadA*-R |  | TTATTTGCCGACTACCTTGGTGATCTC |
| pTarget-F | pACYCDuet-1, vector for constructing pTarget | CCAAGGTAGTCGGCAAATAATGCTTAAGTCGAACAGAAAGTAATCGTATTG |
| pTarget-R |  | ATTTCCTAATGCAGGAGTCGCATAAGG |
| pMuta-wt-F1 | pET28a, vector for constructing pMuta1 | ctaaagaggagaaaagatctatgATGCACCACCACCACCACCAC |
| pMuta-wt-R1 |  | CGTCGCCGCACTTATGACTG |
| sgRNA-F | sgRNA, gene for constructing pMuta1 | GTCATAAGTGCGGCGACGttgacagctagctcagtcctagg |
| sgRNA-R |  | GCGCGGGGCATGACTATcggtggtgataaacttatcatcccct |
| pMuta-wt-F2 | pMuta1, vector for constructing pMuta2 | ATAGTCATGCCCCGCGCC |
| pMuta-wt-R2 |  | cttaaatgtgaaagtgggtcttaaGCGCAACGCAATTAATGTAAGTTAGC |
| TetR-ptet-F | tetracycline-inducible promoter (including its repressor), gene for constructing pMuta2 | ttaagacccactttcacatttaagttgtttttctaatcc |
| TetR-ptet-R |  | CATcatagatcttttctcctctttagatcttttgaattcttttc |
| pMuta-F1 | pMuta2, vector for constructing pMuta-nCas9 | GAGTCAGCTAGGAGGTGACTGAGATCCGGCTGCTAACAAAGC |
| pMuta-R1 |  | CATcatagatcttttctcctctttagatcttttgaattcttttc |
| nCas9-F | nCas9, gene for constructing pMuta-nCas9 | atctaaagaggagaaaagatctatgATGGATAAGAAATACTCAATAGGCTTAGCAATCG |
| nCas9-R |  | GTCACCTCCTAGCTGACTCAAATCAATG |
| pMuta-F2 | pMuta2, vector for constructing pMuta-T5DNAP4M | AACGTATCCTGGGCGCATGAGATCCGGCTGCTAACAAAGCC |
| pMuta-R2 |  | CATcatagatcttttctcctctttagatcttttgaattcttttc |
| T5DNAP4M-F | T5DNAP4M, gene for constructing pMuta-T5DNAP4M | ctaaagaggagaaaagatctatgATGAAGATCGCGGTTGTTGATAAAGC |
| T5DNAP4M-R |  | TGCGCCCAGGATACGTTTTACG |
| pMuta-T5-F | pMuta-T5DNAP4M, vector for constructing pMuta-T5DNAP4M-nCas9 | GTCAGCTAGGAGGTGACTGAGATCCGGCTGCTAACAAAGCC |
| pMuta-T5-R |  | TGCGCCCAGGATACGTTTTACG |
| 23aa-nCas9-F | nCas9, gene for constructing pMuta-T5DNAP4M-nCas9 | AACGTATCCTGGGCGCAggttctagtgaaaccccgggaac |
| 23aa-nCas9-R |  | TCAGTCACCTCCTAGCTGACTCAAATCAATG |
| pMuta-nCas9-F | pMuta-nCas9, vector for constructing pMuta-nCas9-T7DNAP3M | CTGGGCGATCTGCCACTAAGATCCGGCTGCTAACAAAGCC |
| pMuta-nCas9-R |  | GTCACCTCCTAGCTGACTCAAATCAATG |
| 23aa-T7DNAP3M-F | T7DNAP3M, gene for constructing pMuta-nCas9-T7DNAP3M | GATTTGAGTCAGCTAGGAGGTGACggttctagtgaaaccccgggaac |
| 23aa-T7DNAP3M-F |  | TTAGTGGCAGATCGCCCAGTTC |
| pMuta-T5-23aa nCas9-F1 | pMuta-T5DNAP4M-nCas9, vector for constructing pMuta-T5DNAP4M-MCP+nCas9 | gaAAGGAGATATACATATGGATAAGAAATACTCAATAGGCTTAGCAATCG |
| pMuta-T5-23aa- nCas9-R1 |  | GGCCtagctctaaaacTATTAATGTACGGCCAGCAAactagtattatacc |
| M13sg-F | sgRNA containing MS2 hairpin loop, gene for constructing pMuta-T5DNAP4M-MCP+nCas9 or pMuta-nCas9+MCP-T7DNAP3M | GTACATTAATAgttttagagctaGGCCAACATGAGG |
| M13sg-R |  | ccgactcggtgccacttGGCCCTGCAGACATGGG |
| pMuta-T5-23aa-nCas9-F2 | T5DNAP4M, gene for constructing pMuta-T5DNAP4M-MCP+nCas9 | aagtggcaccgagtcgg |
| pMuta-T5-23aa-nCas9-R2 |  | ACTGGGTGAAGTTAGACGCCATggatccgctaccccctgatc |
| MCP-5F | MCP, gene for constructing pMuta-T5DNAP4M-MCP+nCas9 | ATGGCGTCTAACTTCACCCAGTTCG |
| MCP-5R |  | GTATTTCTTATCCATATGTATATCTCCTTtcAGTAGATACCAGAGTTCGCCGC |
| pMuta-nCas9-23aa-T7-F1 | pMuta-nCas9-T7DNAP3M, vector for constructing pMuta-nCas9+MCP-T7DNAP3M | GCGGCGAACTCTGGTATCTACggttctagtgaaaccccgggaac |
| pMuta-nCas9-23aa-T7-23aa-R1 |  | GGCCtagctctaaaacTATTAATGTACGGCCAGCAAactagtattatacc |
| pMuta-nCas9-23aa-T7-F2 | pMuta-nCas9-T7DNAP3M, gene for constructing pMuta-nCas9+MCP-T7DNAP3M | aagtggcaccgagtcgg |
| pMuta-nCas9-23aa-T7-R2 |  | GACGCCATATGTATATCTCCTTtcaGTCACCTCCTAGCTGACTCAAATCAATG |
| MCP-7F | MCP, gene for constructing pMuta-nCas9+MCP-T7DNAP3M | tgaAAGGAGATATACATATGGCGTCTAACTTCACCCAGTTCG |
| MCP-7R |  | GTAGATACCAGAGTTCGCCGC |
| pMuta-T5-23aa nCas9-F3 | pMuta-T5DNAP4M-nCas9, vector for constructing pMuta-T5DNAP4M-SPY+nCas9 | GCGGTCTGCAGATGGATAAGAAATACTCAATAGGCTTAGcaATCG |
| pMuta-T5-23aa nCas9-R3 |  | CAGAGCCACCggatccgctaccccctgatcc |
| Spytag-5F | Spytag, gene for constructing pMuta-T5DNAP4M-SPY+nCas9 | tagcggatccGGTGGCTCTGGTAGCGGC |
| Spytag-5R |  | GGTAATACGAATGTATATCTCCTTTTATTTAGTCGGTTTATACGCATCAACCATCAC |
| Spycatcher-5F | Spycatcher, gene for constructing pMuta-T5DNAP4M-SPY+nCas9 | CCGACTAAATAAAAGGAGATATACATTCGTATTACCATCACCACCACCAC |
| Spycatcher-5R |  | TTCTTATCCATCTGCAGACCGCTACCGC |
| pMuta-nCas9-23aa-T7-F3 | pMuta-nCas9-T7DNAP3M, vector for constructing pMuta-nCas9+SPY-T7DNAP3M | ATGATCGTTTCTGCTATCGCGG |
| pMuta-nCas9-23aa-T7-R3 |  | GTCACCTCCTAGCTGACTCAAATCAATG |
| Spytag-7F | Spytag, gene for constructing pMuta-nCas9+SPY-T7DNAP3M | GATTTGAGTCAGCTAGGAGGTGACGGTGGCTCTGGTAGCGGC |
| Spytag-7R |  | GGTAATACGAATGTATATCTCCTTTTATTTAGTCGGTTTATACGCATCAACCATCAC |
| Spycatcher-7F | Spycatcher, gene for constructing pMuta-nCas9+SPY-T7DNAP3M | CCGACTAAATAAAAGGAGATATACATTCGTATTACCATCACCACCACCAC |
| Spycatcher-7R |  | CGCGATAGCAGAAACGATCATggatccgctaccccctgatc |
| Pt5test-F | pMuta-T5DNAP4M-MCP+nCas9, vector for constructing Pt5test | GATCCGGCTGCTAACAAAGCCC |
| Pt5test-R |  | AACCACCACCACCAGAACCGCCACCAGAACCGTCACCTCCTAGCTGACTCAAATCAATGC |
| EGFP-5F | EGFP, gene for constructing Pt5test | GGTTCTGGTGGTGGTGGTTCTatgggtaagggagaagaacttttcac |
| EGFP-5R |  | GGGCTTTGTTAGCAGCCGGATCttatttgtatagttcatccatgccatgtgtaatc |
| Pt7test-F | pMuta-nCas9+MCP-T7DNAP3M, vector for constructing Pt7test | tgaAAGGAGATATACATATGGCGTCTAACTTCACCCAGTTCG |
| Pt7test-R |  | AACCACCACCACCAGAACCGCCACCAGAACCGTCACCTCCTAGCTGACTCAAATCAATGC |
| EGFP-7F | EGFP, gene for constructing Pt7test | GGTTCTGGTGGTGGTGGTTCTatgggtaagggagaagaacttttcac |
| EGFP-7R |  | GACGCCATATGTATATCTCCTTtcattatttgtatagttcatccatgccatgtgtaatc |
| Pt5/7test-F2 | Pt5/7test, vector for constructing Pt5/7test-dTnpB | CCTCTAAACGGGTCTTGAGGGGTTTTTTGCTGAAACCTCAGGCATTTGAGACGCGCCCTGTAGCG |
| Pt5/7test-R2 |  | ggactgagctagctgtcaaCCCATTCGCCAATCCGGATATAG |
| TnpBgRNA-F | TnpBgRNA, gene for constructing Pt5/7test-dTnpB | GGttgacagctagctcagtcctaggtataatac |
| TnpBgRNA-R |  | CATCTAAAATATACTgtcccattcgccatgccg |
| araC-Para-F | arabinose-inducible promoter (including its repressor), gene for constructing Pt5/7test-dTnpB | catggcgaatgggacAGTATATTTTAGATGAAGATTATTTCTTAATCTAGACATGAGCG |
| araC-Para-R |  | CGCTTTGTTACGGATCATTTTTTATAACCTCCTTAGAGCTCGAATTCCCR |
| dTnpB-F | dTnpB, gene for constructing Pt5/7test-dTnpB | GGAGGTTATAAAAAATGATCCGTAACAAAGCGTTCGTTG |
| dTnpB-R |  | CCTCAAGACCCGTTTAGAGGCCCCAAGGGGTTATGCTAGTTATTGCTCAGCGGTGGCAGCAGTTACACAACCAGGGTAGCGTGG |
| pP43NMK-F1 | pP43NMK, vector for constructing reporter plasmid for *B. subtilis* 168 | gagctgggttttttgtttgttgcttggcgtaatcatggtcatagc |
| pP43NMK-R1 |  | GATCACCGCTTCCCTCATgcataaaatcccctttcattttctaatgtaaatctattacc |
| AadA*-F | aadA with the premature stop codon TAA, gene for constructing reporter plasmid for *B. subtilis* 168 | ATGAGGGAAGCGGTGATCG |
| AadA*-R |  | TTATTTGCCGACTACCTTGGTGATCTC |
| pP43NMK-F2 | pP43NMK, vector for constructing reporter plasmid for *B. subtilis* 168 | CCAAGGTAGTCGGCAAATAAagggaattgatgaattatatcaacatattaagcctttg |
| pP43NMK-R2 |  | atttaaattatatcaacgttaataagtgcgactcaaaaaatctccacct |
| Pveg-sgRNA-F | sgRNA cassette, gene for constructing reporter plasmid for *B. subtilis* 168 | cgcacttattaacgttgatataatttaaattttatttgacaaaaatgggctcgtgttgt |
| Pveg-sgRNA-R |  | aacaaacaaaaaacccagctcattgagc |
| pHT01-F | pHT01, vector for constructing mutator plasmid for *B. subtilis* 168 | TCTAGAGTCGACGTCCCCG |
| pHT01-R |  | GGTACCAAGCTAATTCCGGTGG |
| BS-Ptet-T5/7-F | tetracycline-inducible promoter, gene for constructing mutator plasmid for *B. subtilis* 168 | CACCGGAATTAGCTTGGTACCgatcaataaaaaacagcccgcagatc |
| BS-Ptet-T5/7-R |  | GGTACCAAGCTAATTCCGGTGG |
| pUDP002-5F | pUDP002, vector for constructing KL-T5DNAP4M-MS2 | taatggcagcttttgattaagccttctag |
| pUDP002-5R |  | tccggatcctgggactcc |
| KL-T5-F | T5DNAP4M mutaor, gene for constructing KL-T5DNAP4M-MS2 | ggagtcccaggatccggaAAGATCGCCGTTGTCGATAAGG |
| KL-T5-R |  | tagaaggcttaatcaaaagctgccattattagtcgcctcccagctgag |
| pUDP002-7F1 | pUDP002, vector for constructing KL-T7DNAP3M-MS2+TrxA | cacaggccccttttcctttgt |
| pUDP002-7R1 |  | tccggatcctgggactcc |
| KL-T7-F | T7DNAP3M mutator, gene for constructing T7DNAP3M-MS2+TrxA | ggagtcccaggatccggagacaagaagtacagcatcggcc |
| KL-T7-R |  | aggcttaatcaaaagctgccattattaGTGACAAATAGCCCAGTTTGGACC |
| pUDP002-7F2 | pUDP002, vector for constructing KL-T7DNAP3M-MS2+TrxA | taatggcagcttttgattaagccttctag |
| pUDP002-7R2 |  | tctgaccaatcctttgccgtagt |
| PADH1-TrxA-F | TrxA cassette, gene for constructing KL-T7DNAP3M-MS2+TrxA | acggcaaaggattggtcagaacactgcctcattgatggtgg |
| PADH1-TrxA-R |  | ttccctgtatgaagcgagcgaatttc |
| pUDP002-7F3 | pUDP002, vector for constructing KL-T7DNAP3M-MS2+TrxA | ctcgcttcatacagggaaagttcg |
| pUDP002-7R3 |  | tttgtttgtttatgtgtgtttattcgaaactaagtt |
| PTDH3-sgRNA-F | sgRNA cassette, gene for constructing KL-T5/7DNAP3M-MS2 | gtttcgaataaacacacataaacaaacaaattgaaactgatgagtccgtgagg |
| PTDH3-sgRNA-R |  | acaaaggaaaaggggcctgtgtcccattcgccatgccgaag |

**Table S4. Sequence information of mutations occurring in tatA or tatB genes**

| Mutation | Amino acid changes | Nucleotide changes | Nucleotide number between mutation and the nick |
| --- | --- | --- | --- |
| EvoL1 | —— | 5' untranslated region of tatA, -10:A→G | 1531 |
| EvoL2 | TatA(A76A) | GCT→GCA | 1294 |
| EvoL3 | TatA(A57V) | GCT→GTT | 1352 |
| EvoL4 | TatB(D3G) | GAT→GGT | 1241 |
| EvoL5 | TatB(K68E) | AAG→GAG | 1047 |
| EvoL6 | TatB(A121A) | GCT→GCC | 886 |
